# Molecular rotors reveal the 3D viscous habitat of mucus-colonizing bacteria

**DOI:** 10.1038/s41467-026-75892-y

**Published:** 2026-07-22

**Authors:** Bryce G. Inman, Nirav Patel, Gabriel Castro-Falcón, Taylor Hernandez, Ryan J. McGorty, Aki H. Ohdera, Logan M. Gonzalez, Kay D. Bidle, Farooq Azam, Stuart Humphries

**Affiliations:** 1 Scripps Institution of Oceanography, University of California San Diego, La Jolla, CA USA; 2 Woods Hole Oceanographic Institution, Woods Hole, MA USA; 3 School of Natural Sciences, University of Lincoln, Lincoln, United Kingdom; 4 Department of Physics and Biophysics, University of San Diego, San Diego, CA USA; 5 Department of Mathematics, University of Arizona, Tucson, AZ USA; 6 Department of Marine and Coastal Sciences, Rutgers University, New Brunswick, NJ USA

**Keywords:** Biophysics, Biological physics, Microbial ecology, Marine biology, Confocal microscopy

## Abstract

Interactions between bacteria and particulate organic matter, algae, coral reefs, fish, plant root systems, animals, and humans occur primarily through a dynamic interface of viscous mucus or mucilage. While mucus influences fundamental rates of bacterial infection, respiration, and carbon and nutrient cycling, our observations of this physical habitat of bacteria are limited by methods that damage the material and obfuscate spatial relationships. We present a technique using confocal microscopy of molecular rotors to reveal the 3D viscous structure of undisturbed mucus and associated bacteria. Quantification of the internal viscosity of mucus from different sources highlights variations in microscale morphologies that structure microbial distributions and ecological interactions. Individual examples of mucus aggregates from cultures of *Chaetoceros affinis* and *Pseudo-nitzschia* sp. diatoms exhibit consolidated versus patchy viscous morphologies, respectively, along with distinct patterns of microbial colonization. A viscous mucus layer surrounding an ephyra of the upside-down jellyfish *Cassiopea xamachana* may maintain a local microbiome while preventing direct contact with the animal. By quantifying the complex “mucoscape” shaping bacteria-organic matter interactions, this method provides a physical context for chemical fluxes and microbial activity in diverse ecosystems.

## Abstract (web summary)

Authors quantify the 3D viscous structure of undisturbed mucus and associated bacteria using molecular rotors. Diatom aggregates and the mucus layer of a jellyfish ephyra show distinct viscous morphologies and bacterial colonization patterns.

## Introduction

Mucus – viscous biopolymer matrices such as mucins, mucilage, slimes, and extracellular polymeric substances (EPS) – represents both a food source and physical habitat for bacteria in most ecosystems on the planet. Marine snow is organic detritus made up of polysaccharides and other intracellular components of plankton that aggregate in the upper ocean and sink to depth. Bacteria colonize and degrade viscous marine snow aggregates, recycling nutrients in the water column and regulating the global ocean carbon sink^1–3^. Exopolysaccharides in the viscous phycosphere surrounding phytoplankton act as an interface for two-way exchange of metabolites with bacteria^4–6^. Kelp mucilage (polysaccharides) attracts bacteria that provide a source of reduced nitrogen and vitamins for their host^7,8^. The mucus layers of coral polyps and fish (glycoproteins and polysaccharides) both contain microbiomes that protect their hosts from other pathogens^9–11^. Mucilage in the rhizosphere of plant root systems (polysaccharides) supports microbial assemblages by retaining moisture and providing a source of carbon and other nutrients^12^. Animal and human guts and airways are lined with mucin matrices (glycoproteins), hosting bacteria that aid in digestion and protection from diseases^13–15^. Bacteria can secrete their own EPS that captures micronutrients, provides protection from other organisms and environmental factors, and supports biofilm formation^16,17^. The viscosity of mucus can influence bacterial metabolic activity^18^, nutrient retention, and the flux of molecules between bacteria and other organisms^6^. Therefore, mapping the microscale viscous “mucoscape” provides an important physical context for microbial processes.

Despite the importance of mucus as a habitat for bacteria, the microscale physical structure of undisturbed mucus has never been characterized due to methodological limitations. Abundant transparent exopolymer particles (TEP) were first identified in ocean samples using alcian blue staining, but this method requires filtration and drying steps that flatten and deform the fragile mucus material^19,20^. Other monomer-specific or polymer stains, such as labeled lectins, calcofluor white, or SYPRO Ruby, bind to the exterior surface of mucus, identifying molecular components but no details of the mucus interior^5,21^. Stained thin-sections of mucus preserved in methacrylate or paraffin reveal internal features but modify its natural structure during conversion to a solid phase^7,13^. The NanoSIMS technique provides spatial isotope imaging but also requires filtration and gold-plating^22^. None of these methods allows quantification of a 3D physical parameter in mucus material. Multiple particle tracking microrheology (MPTM) can be used to calculate mucus viscosity as a function of microsphere diffusivity, but the beads do not readily penetrate into thicker mucus, and the technique does not allow localization of bacteria^5^. Topographic imaging using atomic force microscopy (AFM) is also limited to exterior mucus surfaces^23,24^.

To overcome these limitations, we present a method that quantifies the 3D nanoscale viscosity of natural mucus and bacterial associations using confocal microscopy and molecular rotors. Molecular rotors are viscosity-sensing dyes that release energy from photoexcitation either by fluorescing or rotating a functional group of the molecule. As rotation of the functional group is hindered by local viscosity, the fluorescence intensity increases. A variety of molecular rotors have been designed by synthetic chemists primarily for biomedical applications^25,26^. These dyes are typically hydrophobic and have been used to monitor protein conformational changes and the viscosity of cell or organelle membranes^27^. The RY3 molecular rotor is hydrophilic, readily penetrates mucus material, and enables a quantitative measurement of viscosity with ratiometric fluorescence^28^. We have adapted this molecular rotor technique to image mucus colonized by bacteria by improving the calibration and image quality, accounting for autofluorescence and pH in the samples, and counterstaining with SYBR Green to localize bacteria. Here, we characterize variations in the 3D viscous structure and bacterial colonization patterns of individual mucus aggregates from cultures of the diatoms *Chaetoceros affinis* and *Pseudo-nitzschia* sp., and the mucus layer surrounding the ephyra of the upside-down jellyfish *Cassiopea xamachana*. This method can be applied to diverse ecological and biological systems for determining the physical niche space occupied by bacteria.

## Results

**Fig. 1:**
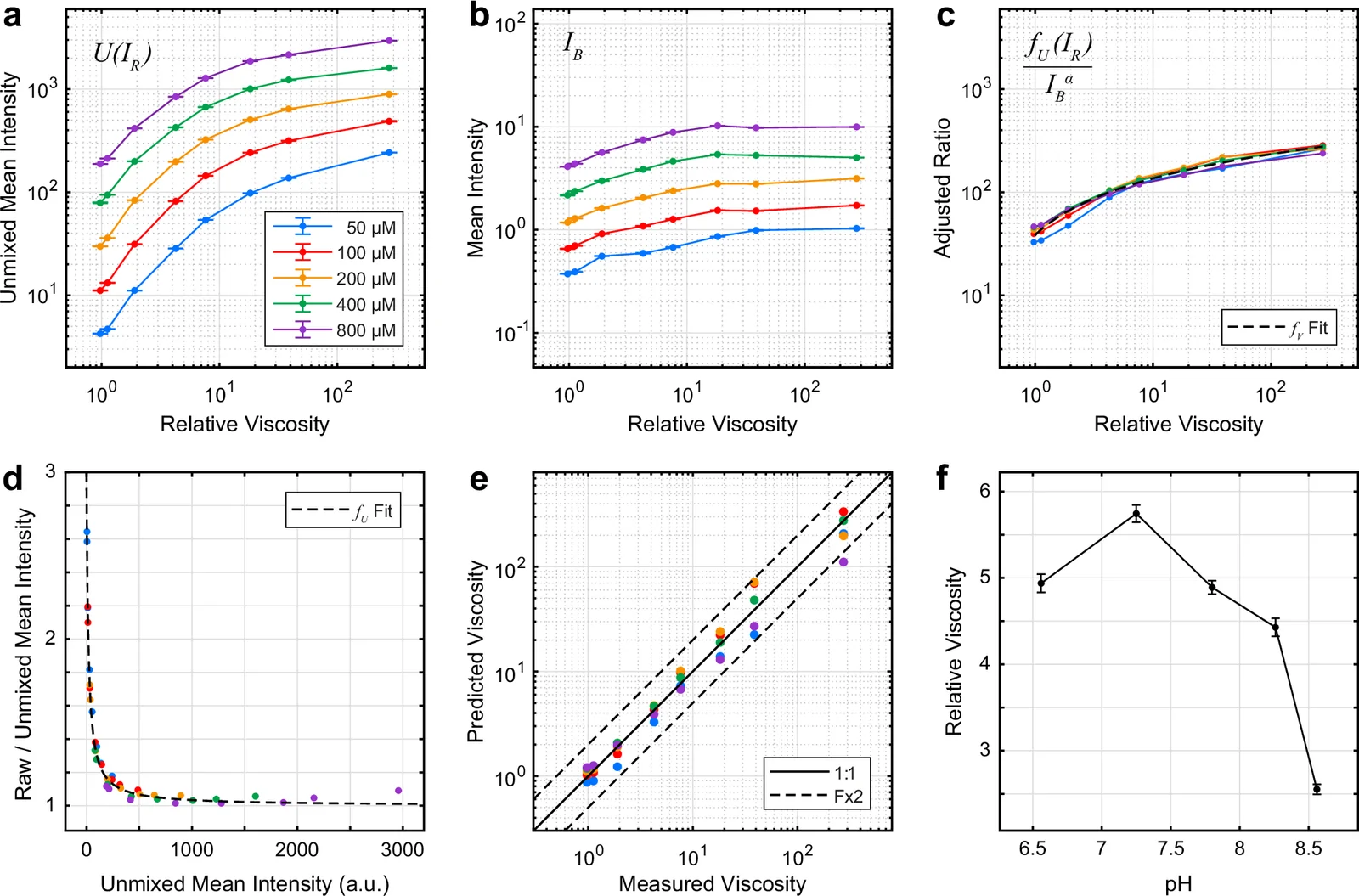
Confocal microscopy calibration of the ratiometric RY3 molecular rotor to viscosity relative to water in solutions of aqueous glycerol. **a** The unmixed red RY3 fluorescence *U*(*I*_*R*_) varies with both viscosity and dye concentration. **b** The raw blue RY3 fluorescence *I*_*B*_ varies more with dye concentration than viscosity. **c** The adjusted ratio of red to blue fluorescence substantially reduces the dependence on dye concentration, providing a quantitative measurement of relative viscosity through the fit of *f*_*V*_ (Eq. 1). **d** After separating the red RY3 fluorescence from chlorophyll autofluorescence, the unmixed red fluorescence is rescaled to raw values via *f*_*U*_ (Eq. 2) to preserve the concentration-independence of the ratio in (**c**). **e** The performance of the calibration is demonstrated by comparing the relative viscosities measured with a rheometer to those predicted by the calibration. Predicted values lying between the dashed lines are within a factor of 2 of the measured viscosities. **f** Variation in the apparent viscosity with pH for solutions of the same glycerol concentration. Error bars in (**a**, **b**, and **f**) are the standard deviation for *n* = 3 technical replicates. Source data for (**a**–**f**) are provided as a Source Data file.

The molecular rotor RY3 produces a quantitative measure of nanoscale viscosity through ratiometric imaging, even if the dye is not uniformly distributed at the microscale in a complex biofluid. For example, if the dye accumulates in certain regions of a mucus aggregate, an increased fluorescence signal may result from either higher dye concentration or higher viscosity, and we cannot differentiate the two with fluorescence intensity alone. This requires calibration of RY3 across standard solutions that vary in both viscosity and dye concentration (Fig. 1). Using ~400 nm excitation, RY3 fluoresces strongly at red wavelengths ~650 nm (which is more sensitive to the local viscosity; Fig. 1a) and weakly in the blue 420–500 nm range (which is more sensitive to the concentration of the rotor; Fig. 1b). The dependence of red fluorescence on dye concentration (Fig. 1a) is removed using blue fluorescence (Fig. 1b) and the new calibration Eq. (1), yielding a single curve that only depends on viscosity (Fig. 1c; see “Methods”). Any autofluorescence due to chlorophyll or other naturally-occurring fluorophores is separated from the red fluorescence through spectral unmixing, which is then rescaled using Eq. (2) to preserve the ratiometric properties of RY3 (Fig. 1d). The performance of the calibration is demonstrated by comparing viscosities of the solutions measured on a rheometer to those measured on the microscope and predicted by the calibration (Fig. 1e). Across a factor of 16 change in dye concentration and a factor of 275 change in viscosity, 98% of the predicted values are within a factor of 2 (dashed lines) of the measured values (solid line; *N* = 40). For relative viscosities less than 10, 84% of the predicted values are within a factor of 1.25 of the measured viscosities (*N* = 25). A relative viscosity difference of 0.1 can be resolved in calibrated images, according to a paired-sample *t* test of the normally-distributed predicted viscosities (paired by dye concentration) comparing measured values of 1.0 and 1.1 (*p*-value < 0.001, *N* = 5 pairs).

Validation of the molecular rotor method in complex, chemically-heterogeneous mucus material is provided in the Supplementary Information (Supplementary Figs. 1–3). In brief, calibrations of RY3 in solutions of varying complexity are all fit by the same power law (Supplementary Fig. 3), demonstrating that the viscosity measurements do not depend on the composition or complexity of the calibration solutions. Further, the relative viscosity probabilities measured in *C. affinis* aggregates with RY3 strongly agree with multiple particle tracking microrheology (MPTM) measurements of aggregates from the same species published in a separate study (Guadayol et al. 2021; Supplementary Fig. 2). These separate lines of evidence establish the reliability of mucus viscosity measurements using RY3. Bacteria can change the pH of water near their cells over a range of − 1.7 to + 0.4 (Sole et al. 2000; Liu et al. 2022), which can influence dye fluorescence. Over a pH range of 6.6 to 8.3 that spans bacterial modification of water or seawater pH, calibrated viscosities vary by less than 15% (Fig. 1f). The addition of buffering agents should be considered in systems where organisms could reduce the pH below 6.6 or increase the pH above 8.3.

### The 3D viscous structure of individual diatom aggregates informs bacterial colonization patterns

**Fig. 2:**
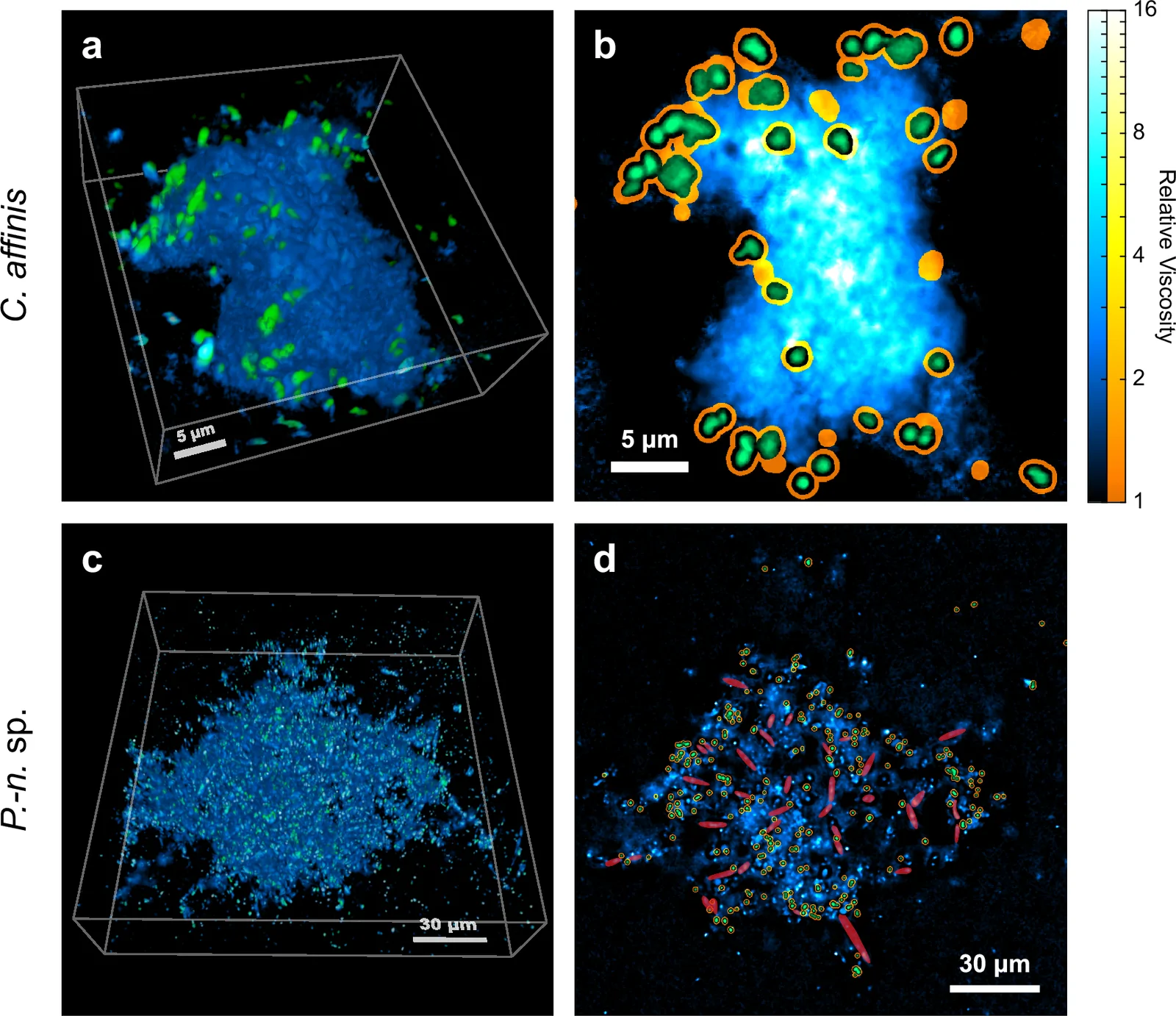
The viscous structure of mucus aggregates differs between the centric diatom *Chaetoceros affinis* (a and b) and the pennate diatom *Pseudo-nitzschia* sp. (c and d). In the composite 3D reconstructions (**a**, **c**) and thin slices (**b**, **d**), green is a nucleic acid stain indicating bacteria, the blue color scale is viscosity relative to background seawater, and the orange color scale highlights viscosities within 0.3 μm of the bacterial cells. The relative viscosity color scale applies to all panels. Phytoplankton cells are absent in the *C. affinis* aggregate but are highlighted in red in the *P.-n*. sp. aggregate slice (**d**).

**Fig. 3:**
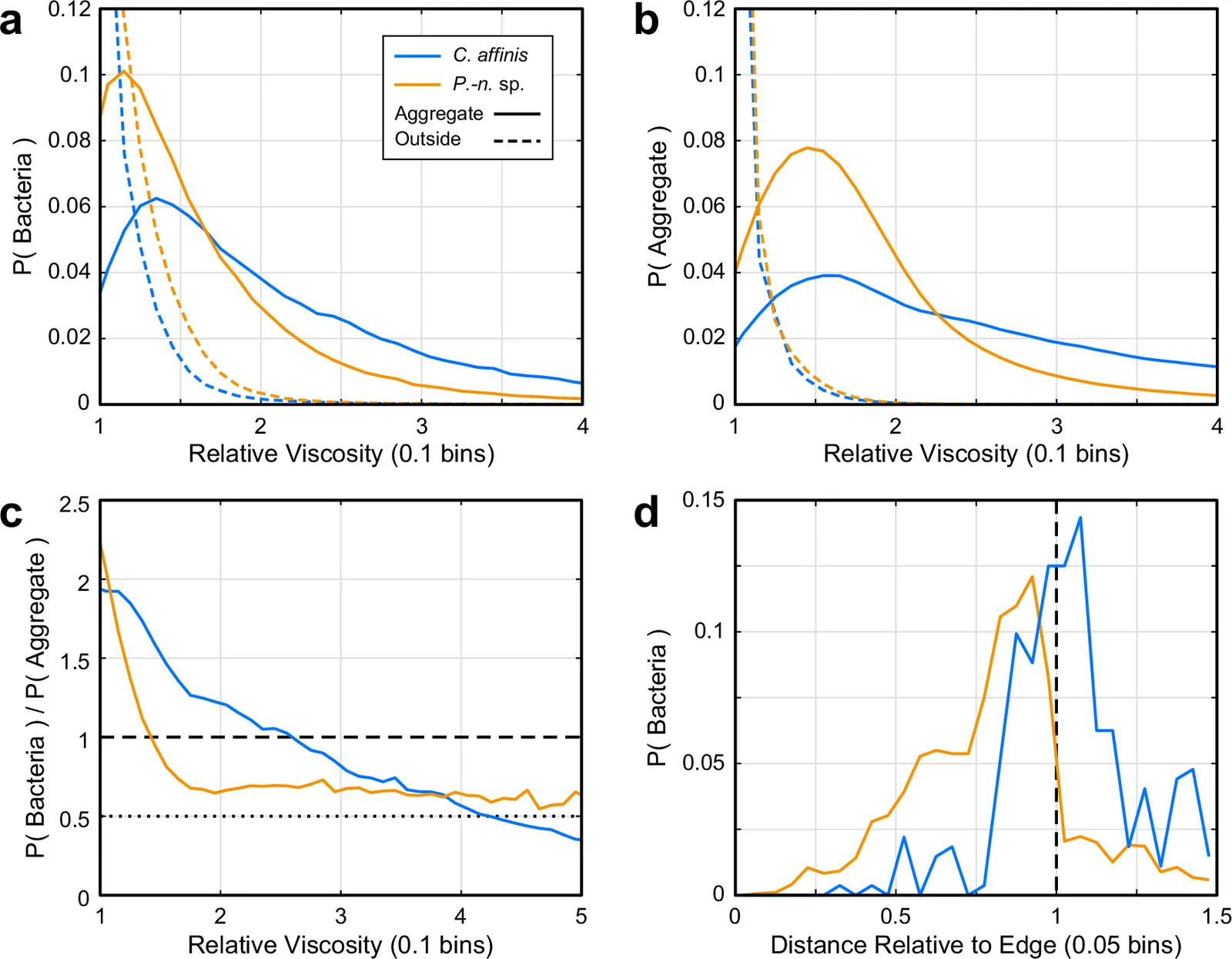
The viscous structure of aggregates in relation to bacterial colonization patterns. **a** The probability (P) of a relative viscosity near bacteria associated with the aggregates (solid lines) versus bacteria outside of the aggregates (dashed lines) shown in Fig. 2. Bacteria colonizing the *C. affinis* aggregate and the *P.-n*. sp. aggregate are most associated with viscosities of 1.35 and 1.15, respectively. Free-living bacteria outside the aggregates are most associated with a viscosity of 1 (seawater). **b** The probability of a relative viscosity inside (solid lines) or outside (dashed lines) of the aggregates. The most probable viscosities in the *C. affinis* and *P.-n*. sp. aggregates are 1.55 and 1.45, respectively. The most probable viscosities outside the aggregates are 1. **c** The ratio of viscosities near bacteria versus the rest of the aggregate. Bacteria are less associated with viscosities below a ratio of 1 (black dashed line). **d** The probability of a bacterium occurring at a relative distance from the center to the edge of the aggregate. A relative distance of 1 (dashed line) is the outer edge of the aggregate, < 1 is inside the aggregate, and > 1 is outside the aggregate. Source data for (**a**–**d**) are provided as a Source Data file.

Aggregates derived from laboratory cultures of the centric diatom *C. affinis* and the pennate diatom *P.-n*. sp. – proxies for marine snow – both exhibit substantial viscous heterogeneity at the nanoscale (Fig. 2). However, the internal viscous structure and pattern of bacterial colonization differ between the two sources. These patterns are consistent across aggregates from each culture, but we focus our analysis on two example aggregates to showcase the utility of the method for extracting quantitative data from individual mucus samples. The *C. affinis* aggregate is small and consolidated with viscosity relative to seawater increasing toward the center (Fig. 2b). No diatom cells are present, and bacteria are located around the edges of the aggregate. The morphology of the *P.-n*. sp. aggregate is larger, patchier, and held together by a lattice of pennate diatom cells (red, Fig. 2d). Bacteria are found around the edges and throughout the interior of the aggregate. While viscosities up to a factor of 16 greater than seawater are present in both aggregates, higher viscosities are more common in the *C. affinis* aggregate as indicated by the skewed distribution in Fig. 3b. The most probable viscosity is 1.55 for the *C. affinis* aggregate and 1.45 for the *P.-n*. sp. aggregate (the peaks of the probability curves; solid lines), compared to 1 for the surrounding seawater (dashed lines). Aggregate bacteria are mostly associated with a viscosity of 1.35 for *C. affinis* (*N* = 1 aggregate, *N* = 518 bacteria) and 1.15 for *P.-n*. sp. (*N* = 1 aggregate, *N* = 6972 bacteria, Fig. 3a), which are lower than the most probable viscosities in their corresponding aggregates. Therefore, the 1.15–1.35 range of viscosities correlates to ‘surface’ regions of the aggregate, which are accessible to bacteria. The interior of the *P.-n*. sp. aggregate is riddled with these surfaces, whereas they are only found around the exterior of the *C. affinis* aggregate.

The ratio of viscosity probabilities near bacteria (orange color scale Fig. 2) versus the rest of the aggregates (blue color scale Fig. 2) further characterizes the relationship between bacteria and the viscous structure of aggregates (Fig. 3c). A probability ratio greater than 1 indicates a preferential association of the bacteria with an aggregate viscosity, and a probability ratio less than 1 indicates viscosities that are less accessible to bacteria (dashed line). Bacteria are half as likely to encounter a viscosity with a probability ratio of 0.5, correlating to a physical ‘barrier’ to infiltration (dotted line). Bacteria colonizing the *C. affinis* aggregate are less associated with viscosities greater than ~ 2.5 and are not typically found inside viscosities greater than ~ 4.0 toward the center of the aggregate. While the bacteria colonizing the *P.-n*. sp. aggregate are less associated with viscosities greater than ~ 1.5, there are no contiguous high viscosity regions that represent a barrier (the ratio does not cross the 0.5 line), and they can physically access and infiltrate most of the interior of the aggregate.

Imaging the viscous structure of undisturbed aggregates using molecular rotors allows direct quantification of the 3D spatial distribution of bacteria colonizing aggregates with complex morphologies. The spatial arrangement of bacteria is calculated here as the distance from the centroid of the aggregate to each bacterium divided by the distance from the centroid to the edge of the aggregate in the same direction (Fig. 3d). A bacterium with a relative distance of 1 (dashed line) is located on the outer edge of the concave hull of the aggregate. The probability of bacteria occurring inside the aggregate is substantially higher for *P.-n*. sp. than *C. affinis*, with 77% versus 31% of bacteria located within a relative distance of 0.95, respectively. The median relative distance for bacteria on the *C. affinis* aggregate is 1.0 (compared to 0.83 for *P.-n*. sp.), consistent with colonization of the exterior surface.

Differences between the consolidated and patchy aggregate morphologies can be quantified as porosity (the ratio of void to total volume): 0.03 for *C. affinis* and 0.65 for *P.-n*. sp. The low-porosity morphology of the consolidated *C. affinis* aggregate limits bacterial colonization to the exterior surfaces of viscosity ~ 1.35, with internal viscosities > 2.5 correlating to a physical barrier to bacterial infiltration. Bacteria may then penetrate the aggregate over time by reducing the viscosity, presumably through enzyme activity. In contrast, the high-porosity morphology of the patchy *P.-n*. sp. aggregate provides substantial interior surfaces for bacteria colonization, potentially resulting in a high degree of infiltration. Bacteria may localize to the aggregate interior by random encounter, chemotaxis, or by colonizing smaller constituent particles before the aggregate formed. Regardless of the formation or colonization mechanisms, a patchy aggregate provides more surface area for bacterial attachment and activity than a consolidated aggregate of the same volume. Therefore, we expect that patchy aggregates exhibit higher remineralization rates than consolidated aggregates of the same size, all else equal. Further, a qualitative comparison of the internal structure of these two aggregates suggests that the smallest regions free from bacteria are similar in size to the smallest dimension of their source diatom cells, 10 μm for *C. affinis* and 2 μm for *P.-n*. sp. It stands to reason that aggregates built from exopolymers and lysed phytoplankton cells have a fundamental length scale proportional to the size of the source cells that always excludes bacteria, until further degradation takes place.

### A mucus layer may protect an upside-down jellyfish from bacterial contact

**Fig. 4:**
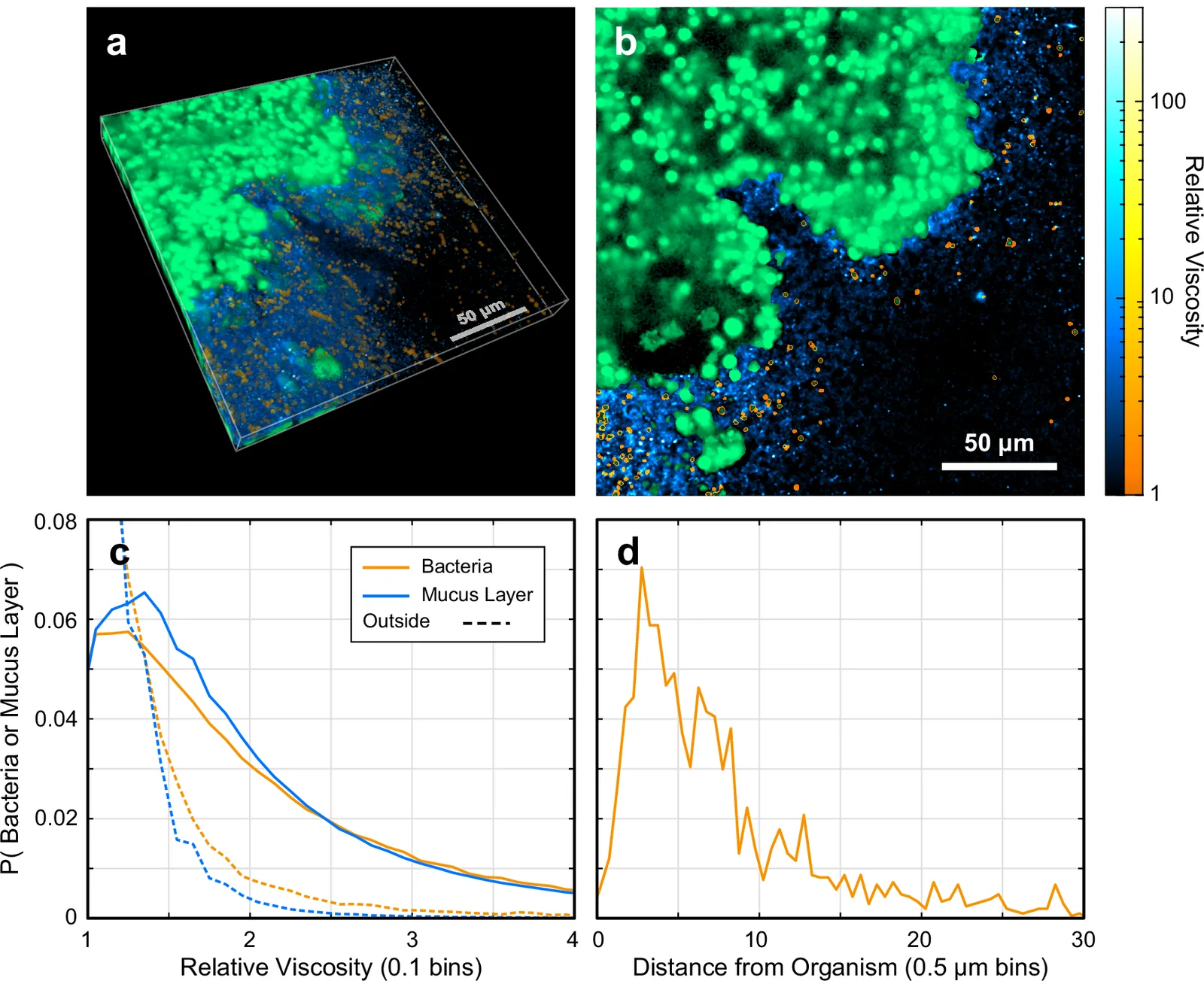
Bacteria in the mucus layer of an upside-down jellyfish ephyra (*Cassiopea xamachana*). A composite 3D reconstruction (**a**) and thin slice (**b**) where green is a nucleic acid stain highlighting the tissue of the jellyfish and bacteria (~ 1 μm) embedded in viscous mucus (blue color scale) exuded by the jellyfish. The orange color scale shows viscosities within 0.3 μm of the bacterial cells and is included in the 3D view to make the bacteria visible. **c** The probability (P) of a relative viscosity near bacteria (solid orange) or in the rest of the mucus layer (solid blue) versus outside the mucus layer (dashed). The most probable viscosity near bacteria in the mucus layer is 1.25 (compared to 1 for bacteria outside the mucus layer). The most probable viscosity in the mucus layer itself is 1.35 (compared to 1 outside the mucus layer). **d** The probability of finding a bacterium at a given distance from the surface of the ephyra in microns. Source data for (**c**, **d**) are provided as a Source Data file.

The upside-down jellyfish *Cassiopea xamachana* produces a mucus layer that hosts a microbiome dominated by Gammaproteobacteria, Bacteroidia, and Alphaproteobacteria^29,30^. The mucus also exhibits specific antimicrobial properties^31^ and contains cassiosome stinging-cell structures used for prey capture^32^. The mucus layer surrounding a live (anesthetized with MgCl), 2 mm-diameter *C. xamachana* ephyra is ~ 50 μm thick (Fig. 4a, Supplementary Movie 1). This layer is characterized by ~ 1-2 μm granules with viscosities up to a factor of ~ 250 greater than seawater embedded in a low viscosity matrix (Fig. 4b). The size of these high viscosity granules is consistent with intracellular mucin granules produced by epidermal mucocytes in corals and other organisms^33,34^, and represents the smallest length scale which can exclude bacteria throughout the layer. The mucus layer has a relative viscosity of 2–10 within 5–10 μm of healthy epidermal cells (upper right, Fig. 4b) and becomes more diffuse farther away, with a most probable viscosity of 1.35 throughout the layer (Fig. 4c). This is lower than the most probable viscosities in the diatom aggregates (Fig. 3a), despite the presence of granules with viscosities an order of magnitude greater than any found in the aggregates. Damage is evident on the lower left of the ephyra (Fig. 4b), presumably with leakage of the mesoglea resulting in increased viscosities farther from the organism.

Bacteria are spread throughout the mucus layer except for in the more viscous material close to the epidermal cells. Bacteria are most associated with viscosities of 1.25 (*N* = 2481, Fig. 4a), similar to those colonizing the diatom aggregates (Fig. 3a), and not the high-viscosity granules. Away from the ephyra, the granules may serve as diffusive nodes producing and linking less viscous material, which bacteria can readily infiltrate. Note that the apparent ~ 10 μm ‘chains’ of bacteria (orange; Fig. 4a) are a result of the jellyfish slowly expanding its body during the 6-minute 3D image capture, pushing both mucus and embedded bacteria outward by ~ 10 μm. The presence of these chains up to 50 μm from the ephyra demonstrates that the mucus layer behaves like a contiguous matrix at larger scales. Bacteria are mostly absent at the surface of the ephyra and increase in abundance to a maximum 3 μm from the organism (Fig. 4d). These observations suggest that the mucus layer prevents potentially harmful bacteria from reaching the surface of the ephyra while keeping the rest of the microbiome relatively close for exchange of chemicals through diffusion. Because the mucus is most viscous close to the organism and then diffuses away or degrades with distance, mucocytes may produce mucus at a rate that maintains this small separation distance with bacteria in addition to other functions such as prey capture.

## Discussion

Microbes interact with most organisms and particulate organic matter through an interface of viscous material. Confocal microscopy of molecular rotors enables quantification of the internal viscous structure of natural, undisturbed mucus. Furthermore, localization of bacteria on and within complex mucus morphologies presents new opportunities for understanding the function of mucus as a physical barrier or interactive habitat for microbial processes. We show that mucus from different sources exhibits unique morphologies that may influence bacterial colonization patterns. A consolidated *C. affinis* aggregate increases in viscosity toward the center, limiting bacterial colonization to the exterior surface. In contrast, a patchy *P.-n*. sp. aggregate held together by diatom cells allows bacteria to access interior surfaces. The mucus layer surrounding a *C. xamachana* ephyra is composed of high-viscosity granules held together by low-viscosity regions, maintaining a microbiome near the organism while preventing direct contact. It is important to note that these relationships between bacterial colonization patterns and mucus viscosities are correlative. Other factors, such as binding of surface-expressed lectins, bacterial motility, or elastic properties of the mucus, may influence bacterial colonization, and further research is needed to determine a causal relationship. The method presented here measures microviscosity at the scale of the molecular rotor; this is not the same as hydrodynamic viscosity measured with a rheometer and does not include information about the elasticity of the fluid. However, these examples provide the first evidence of divergent mucus morphologies creating physical niches occupied by bacteria, paving the way for future research on microbial adaptations for exploiting different mucus structures.

The molecular rotor method can be used to interrogate bacterial interactions with mucus and host organisms in a variety of ecosystems, such as marine snow, kelp forests, coral reefs, land plant root systems, and animal and human respiratory and gut mucosa. The technique can monitor viral or bacterial penetration of protective mucus layers and subsequent infection of animal tissues, with implications for human health and disease control^35^. Paired with metabolomics assays, the molecular rotor method could quantify the conversion of gel-phase organic matter to dissolved solutes that support the rhizosphere of plants or marine food webs. Combining the method with functional proteomics could link specific bacterial enzyme activity to the physical degradation of mucus structure. Because viscosity slows the diffusion of other biologically significant molecules^6^, the 3D morphology of mucus can alter the flow of nutrients between bacteria and host organisms. By revealing the viscous habitat of bacteria, this method provides a physical context for chemical fluxes and biological responses at the microscale.

## Methods

### Ethics statement

Use of *Cassiopea xamachana* jellyfish ephyrae in experimental studies does not currently require ethical approval or permitting, as per the United States Animal Welfare Act USC 2132(g) and the Institutional Animal Care and Use Committee. We endeavored to minimize harm of the jellyfish and adhere to ethical best practices by performing experiments on a minimal number of whole organisms.

### Molecular rotor

The RY3 molecular rotor is hydrophilic, relatively small (~ 500 g mol^−1^), highly photostable, and rapidly permeates mucus^28^. Both local viscosity and dye concentration contribute to the fluorescence intensity of molecular rotors. Because dyes may not be evenly distributed in a complex biofluid such as mucus, ratiometric calibration is required for a quantitative viscosity measurement. Using ~ 400 nm excitation, RY3 fluoresces weakly in the blue 420–500 nm range (which is more sensitive to the concentration of the rotor; Fig. 1b) and strongly at red ~ 650 nm (which is more sensitive to the local viscosity; Fig. 1a). The ratio of the red to the blue fluorescence intensity signal can be calibrated to viscosity as described below. Fluorescence lifetime imaging microscopy (FLIM) of RY3 can also yield a concentration-independent viscosity measurement, but because FLIM instruments are more expensive and relatively rare, we focus on ratiometric measurements (see Supplementary Information). RY3 is not yet commercially available and must be synthesized (contact the authors of this work for options). As such, toxicological data is not currently available, and caution should be taken when handling the dye.

RY3 can be synthesized according to the protocol in Peng et al. (2011) with a molecular weight of 475.06 or 566.53 g mol^−1^ depending on whether chloride or iodide, respectively, is used as the counter ion during synthesis. It is easier to perform the synthesis with iodide salts, but iodide dissociated from RY3 can be harmful to microbes. We recommend replacing the I- with Cl- using an ion exchange column during synthesis. RY3 powder is dissolved in DMSO to make a stock solution with a concentration of 10 mM. The stock is then diluted with pure water or salt water to make 1 mM working solutions. Both the RY3 powder and stock solutions are stored in opaque containers in a − 20 °C freezer.

### Viscosity standards

A series of viscosity standards are used to calibrate the molecular rotor fluorescence ratio to macroviscosity measured with a traditional rheometer. We recommend using glycerol because it exhibits a high degree of molecular crowding and yields the greatest correlation between microviscosity (measured with molecular rotors) and macroviscosity (measured with a rheometer). Additional calibrations with Ficoll and dextran viscosity standards are shown in the Supplementary Information (Supplementary Fig. 3). The viscosities of the standards increase exponentially from water (~ 1 mPa·s) to greater than the highest viscosity expected in experimental samples, so that low microviscosities typical of mucus are better resolved. Standard stocks were prepared by adding glycerol to Milli-Q water with final concentrations of 0, 5, 22, 42, 54, 69, 80, and 99%. The macroviscosities of these standards were measured using a TA Instruments DHR-3 rheometer fitted with a Peltier Concentric Cylinder set to 22 °C laboratory temperature and a Double Gap geometry that is more accurate at low viscosities. Setting the rheometer to perform a logarithmic “flow sweep” from 1 to 100 s^−1^, linear fits of the stresses to the shear rates yielded dynamic viscosities of 1.05, 1.17, 2.15, 5.32, 10.40, 28.86, 75.41, and 908.36 mPa·s via the TA Instruments TRIOS v5.11.0.31 software.

### Calibration of molecular rotor microscopy

Calibration of rotor fluorescence intensity to viscosity requires microscopic imaging of standard samples that vary in both viscosity and dye concentration. The goal is to use the red and blue RY3 fluorescence signals from the sample images to parameterize a function that varies with viscosity and not dye concentration. It is also important to account for any autofluorescence of the experimental subject (e.g., chlorophyll, GFP, etc.) and the optical configuration of the microscope (e.g., choice of objective, laser power, gain) in the calibration. These calibration considerations are summarized as

1 $$\eta={f}_{V}\left(\frac{{f}_{U}\left({I}_{R}\right)}{{{I}_{B}}^{\alpha }}\right)$$

where *η* is the viscosity relative to water, *I*_*R*_ is the red fluorescence intensity of RY3, *I*_*B*_ is the blue fluorescence intensity of RY3, and *α* is a tuning parameter that improves the calibration performance. The function *f*_*U*_ accounts for the autofluorescence signal that is separated from *I*_*R*_ through spectral unmixing. The function *f*_*V*_ is an empirical fit of the fluorescence ratio to the macroviscosity of the standards for a given optical configuration. Using 404 nm excitation, the microscopy calibration performed best by collecting *I*_*R*_ red wavelengths centered on 655 nm and *I*_*B*_ blue wavelengths in the range 420–450 nm (see Supplementary Information). The *I*_*B*_ fluorescence intensity is two orders of magnitude weaker than *I*_*R,*_ and the challenge of capturing both signals at the same time is the primary consideration in optimizing the optical configuration of the microscope. We found that using a relatively high dye concentration of 50 μM in experimental samples helps to offset the low quantum efficiency of RY3 at blue wavelengths without dramatically increasing the laser power required.

#### Calibration sample preparation

Theoretically, ratiometric fluorophores do not require calibration across different dye concentrations because the ratio accounts for variability in emission caused by laser intensity, concentration, and instrument-specific factors^27^. In practice, weak emission peaks and the potential for high dye accumulation in experimental samples make testing a range of dye concentrations a necessity for quantitative calibration. Samples from the viscosity standards are combined with sub-stocks of dye to produce final concentrations of 50, 100, 200, 400, and 800 μM RY3. We do not include dye concentrations < 50 μM because they do not yield enough *I*_*B*_ blue fluorescence for calibration when paired with low viscosity standards on our instrument. We have mostly observed accumulation of dye in certain regions of mucus, but rarely exclusion of dye. Further, when dye accumulates in mucus, the concomitant reduction of dye concentration in the much larger volume of the surrounding fluid is negligible. Therefore, it is more important to account for local increases rather than local decreases relative to the 50 μM bulk dye concentration in an experimental sample. High concentrations of dye ≤ 800 μM do not change the photophysics of RY3 (Supplementary Fig. 8).

To keep the dilution of the viscosity standards constant across the five dye concentrations tested, five separate sub-stocks of dye are prepared as follows. The 10 mM stock of RY3 is diluted with DMSO in a ratio of 1:15 for 50 μM, 1:7 for 100 μM, 1:3 for 200 μM, 1:1 for 400 μM, and no dilution for 800 μM sub-stocks. Adding 16 μL of each sub-stock to 184 μL of each viscosity standard yields forty 200 μL samples for calibration imaging. To account for the 0.92 factor dilution of the viscosity standards, the exponential fit of macroviscosity to glycerol concentration is used to estimate final calibration sample viscosities of 1.00, 1.12, 1.90, 4.26, 7.63, 18.25, 38.68, and 275.33 relative to water. The calibration is only valid within this range. The calibration samples are then pipetted into 8-well chambered coverglass slides (Thermo Scientific 155411), the same used for imaging experimental samples. SYBR Green is also added to experimental samples, but is not necessary for calibration because there is negligible overlap between RY3 and SYBR Green absorption and emission spectra.

#### Calibration microscopy

Optical configuration settings will vary with different confocal microscopes, and a full set of calibration images should be acquired for each optical configuration that is used (e.g., switching objectives or changing gains; see Supplementary Information). We performed our calibration and experiments on an inverted Nikon A1R + confocal microscope with a 4-channel standard detector, 32-channel spectral detector, and NIS-Elements AR 5.21.02 acquisition software. To resolve ~1 μm bacteria and ~ 100 μm mucus aggregates at the same time, we used a x60/1.2 NA water-immersion objective (Nikon MRD07602). After testing various combinations of optical configurations, we determined that the following parameters worked best for imaging mucus viscosity and bacterial associations: 10% power 100 mW 404 nm laser, 1% power 200 mW 488 nm laser, 1.1 μs pixel dwell time, 140–160 gain, and 4x line averaging. Line averaging is the most useful parameter for capturing both strong *I*_*R*_ and weak *I*_*B*_ fluorescence in the same image. A single 1024 × 1024 pixel image is acquired in ~ 8 s with these settings.

Each sample and optical configuration are imaged in triplicate 10 μm above the glass bottom of the chamber. Refraction confounds imaging near the glass bottom, and self-shading can reduce signal far from the bottom because higher dye concentrations are fairly opaque; imaging 10 μm above the chamber bottom avoids these issues and produces consistent results. The vertical location of the slide bottom should be determined prior to imaging each sample, as the distance between the objective lens and the bottom of the slide can vary substantially between chambers. Within a sample, potential photobleaching is minimized by moving the stage horizontally between each image capture such that the images do not overlap.

#### Accounting for autofluorescence with spectral unmixing

Autofluorescence of naturally occurring compounds such as chlorophyll or GFP within mucus or cells can overlap with the RY3 signal and lead to unreliable results when calibrating experimental images. Overlapping fluorescence signals can be separated using a linear spectral unmixing function included in most confocal acquisition software. First, each source of fluorescence is imaged separately with the chosen optical configuration and all relevant detection channels (e.g., SYBR Green, RY3, and chlorophyll *a* with 404 and 488 nm illumination). The individual emission spectra are isolated within the images and saved for unmixing subsequent experimental images. Spectral unmixing works well with the strong *I*_*R*_ signal but reduces the weak *I*_*B*_ fluorescence to undetectable levels. Unmixing *I*_*R*_ but not *I*_*B*_ changes the *I*_*R*_/*I*_*B*_ ratio in a manner that increases concentration dependence and worsens the calibration. Fortunately, this can be corrected by rescaling the unmixed *U*(*I*_*R*_) from calibration images to their raw *I*_*R*_ counterparts with the function

2 $${f}_{U}\left({I}_{R}\right)=U\left({I}_{R}\right)\left(1+\frac{a}{U\left({I}_{R}\right)+b}\right)$$

where *U* is the unmixing algorithm, and the parameters *a* and *b* result from an empirical fit of *U*(*I*_*R*_) to *I*_*R*_/*U*(*I*_*R*_) of the form 1+*a*/(*x* + *b*) (Fig. 1d). This fit gives a preferential weighting to lower *I*_*R*_ values; other fits may be more suitable for different confocal microscopes and unmixing algorithms. It is important to ensure a high signal-to-noise ratio in experimental images (e.g., using line averaging) for accurate spectral unmixing. Autofluorescence and unmixing spectra are available in the Supplementary Information (Supplementary Fig. 4).

#### Ratiometric calibration

The ultimate goal of the calibration is to demonstrate concentration independence by collapsing the *I*_*R*_ versus viscosity curves that are separated by dye concentration onto a single curve using the ratio of emissions and the tuning parameter *α* (Fig. 1c). The adjusted ratios are related to macroscale relative viscosities in Eq. (1) via

3 $${f}_{V}\left(\lambda \right)={{\mbox{e}}}^{c\lambda+d}$$

where *λ* = *f*_*U*_(*I*_*R*_)/*I*_*B*_^*α*^, and *c* and *d* are parameters fitting ln(*λ*) to ln(*η*) with the form *c*·exp(*x*)+*d*. Equation (3) allows small viscosity differences near water to be resolved, and logarithmic fitting improves calibration performance for low viscosities (Fig. 1c). A simple metric for the calibration performance is the sum of the absolute normalized differences between the measured viscosities and those predicted by running *I*_*R*_ and *I*_*B*_ from calibration samples through Eq. (1). The tuning parameter *α* = 1.1 was determined by minimizing this metric.

### Imaging mucus viscosity and associated bacteria

#### Experimental sample preparation and microscopy

To prepare an experimental sample for microscopy, the sample of interest is gently pipetted into a microcentrifuge tube, followed by RY3 stock to a final concentration of 50 μM, and then SYBR Green I to a final dilution of 1.5x. For example, 283 μL of diatom culture (see Supplementary Information for growth conditions) are combined with 15 μL of 1 mM RY3 stock and 2 μL of 100x SYBR Green I stock to yield a 300 μL sample. After gently inverting to mix, the sample is left in the dark for 30 min to allow SYBR Green I to bind to nucleic acids. At least two viscosity standards are prepared in the same manner as the calibration (see Calibration sample preparation) for imaging alongside the experimental samples. These standards serve as a control between imaging sessions and should span the range of viscosities expected in the experimental sample (e.g., water and glycerol with a relative viscosity 20). All experimental samples and standards are carefully pipetted into the same type of chambered coverglass slides used in the calibration. When imaging aggregates on the confocal microscope, each plane of the z-stack should include enough negative space (background fluid with no objects covering at least 25% of the image area) to use as a reference during image processing. Z-stacks are acquired at least 3 μm away from the bottom to avoid optical artifacts and with at most 0.5 μm vertical separation between planes to resolve bacteria. The viscosity standards are imaged 10 μm above the floor of the slide as during the calibration.

#### Experimental image processing and analysis

Experimental images^36^ are first spectrally unmixed to separate the red *I*_*R*_ and SYBR Green I signals from autofluorescence such as chlorophyll *a*. The raw blue *I*_*B*_ and unmixed *I*_*R*_ and SYBR Green I channels are then deconvolved with the point spread function (PSF) of the microscope to improve image quality and reduce 3D optical blur. Some confocal microscope software offers a deconvolution function with a synthetic (estimated) PSF, but the PSF can also be measured experimentally for better deblurring performance (see Supplementary Information). We averaged 16 z-stacks of 0.1 μm Fluoro-Max Red particles (Thermo Scientific R100) suspended in an aqueous 25% Pluronic solution (Sigma-Aldrich P2443) in the chambered coverglass volume to obtain a final PSF. The PSF is downsampled to match the resolution of a given image set and then deconvolved using a Lucy-Richardson algorithm with 3-5 iterations to preserve fine structure near the aggregate. A median filter is then applied to the resulting red *I*_*R*_ and SYBR Green I images to decrease spatial noise. A Gaussian filter is used for the deconvolved blue *I*_*B*_ images to reduce noise and divide-by-zero errors during calibration.

For each 1024 × 1024 pixel plane of a z-stack, a background region of ~ 200 × 200 pixels is selected that is free from objects (mucus, bacteria, etc.). Red and blue channel values smaller than the mean of their respective backgrounds are replaced with those means to prevent small denominator “blowups” and divide-by-zero errors, which occur in the background regions. The red and blue channels are run through the calibration in Eq. (1), and then each resulting plane is divided by its mean background value to obtain viscosity relative to background. The relative viscosities are then scaled by the ratio of the calibrated viscosities of the two standards measured alongside the experimental samples. The calibration, background normalization, and scaling to standards account for variations in dye concentration, background seawater viscosity, temperature, laser intensity, and other environmental and optical artifacts that may occur between imaging sessions. Further image analysis of bacterial locations and aggregate boundaries, as well as culturing conditions are discussed in the Supplementary Information. The calibration and analysis codes^37^ are written in MATLAB (R2024b) and use the MATLAB Image Processing Toolbox v24.2, MATLAB Curve Fitting Toolbox v24.2, Bio-Formats MATLAB Toolbox v8.3.0, and MATLAB bwdistsc v.1.8.0.

### Reporting summary

Further information on research design is available in the Nature Portfolio Reporting Summary linked to this article.

## Supplementary information

Supplementary Information

Description of Additional Supplementary Files

Supplementary Movie 1

Reporting Summary

Transparent Peer Review file

## Source data

Source Data

## Data Availability

The microscopy data generated in this study have been deposited in Dryad at 10.5061/dryad.x3ffbg818 [10.5061/dryad.x3ffbg818]. Unless otherwise stated, all data supporting the results of this study can be found in the article, supplementary, and source data files. Source data are provided in this paper.

## Code availability

Calibration and analysis codes are available in a GitHub repository at 10.5281/zenodo.20800237 [10.5281/zenodo.20800237].
