# Supplementary material for: Molecular rotors reveal the 3D viscous habitat of mucus-colonizing bacteria
Source: Nat Commun. 2026 Jul 22;17:8952. doi: 10.1038/s41467-026-75892-y (PMC13503797; doi:10.1038/s41467-026-75892-y)
Supplement: Supplementary file 1 [file 41467_2026_75892_MOESM1_ESM.pdf]

Supplementary Information

Supplementary Figures

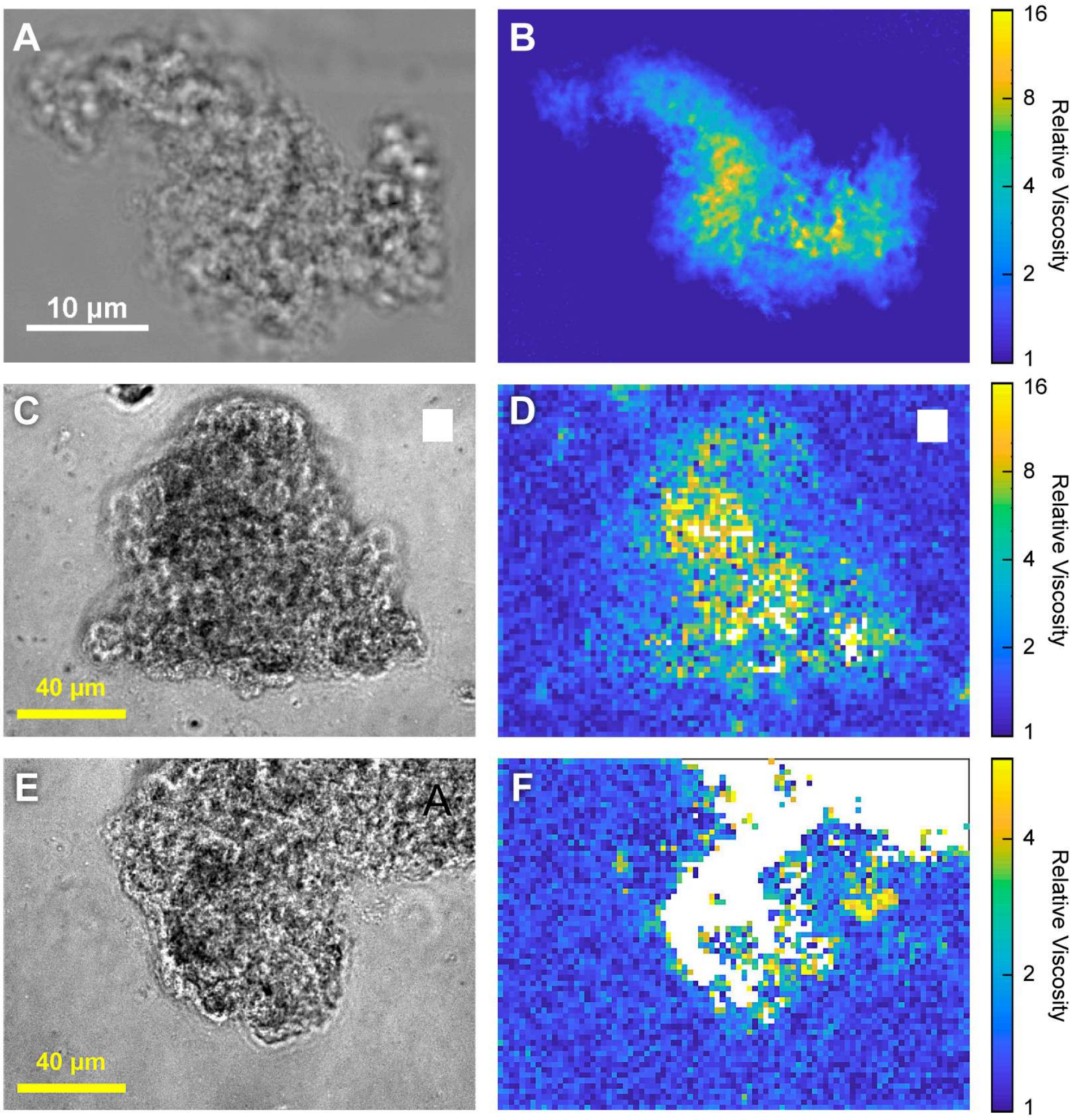

Supplementary Figure 1. **Comparison of *C. affinis* aggregates measured with the molecular rotor RY3 and MPTM.** Transmitted light (**A**) and relative viscosity via RY3 (**B**) from this study. Phase contrast (**C**) and relative viscosity via MPTM (**D**) in a focal plane close to the edge of the aggregate in the z-direction). White pixels are no data, indicating microspheres stuck in the mucus. (**E** and **F**) The same as (C and D) but for a different aggregate showing typical microsphere penetration ~1/3 of the way into the aggregate. Both studies used the same culturing media and similar culturing conditions. The images in (C-F) are adapted from Guadayol et al. (2020) in accordance with the CC BY 4.0 license [https://creativecommons.org/licenses/by/4.0/] with the following changes: the panel label letters have been replaced and the colorbars re-oriented to match the current figure.

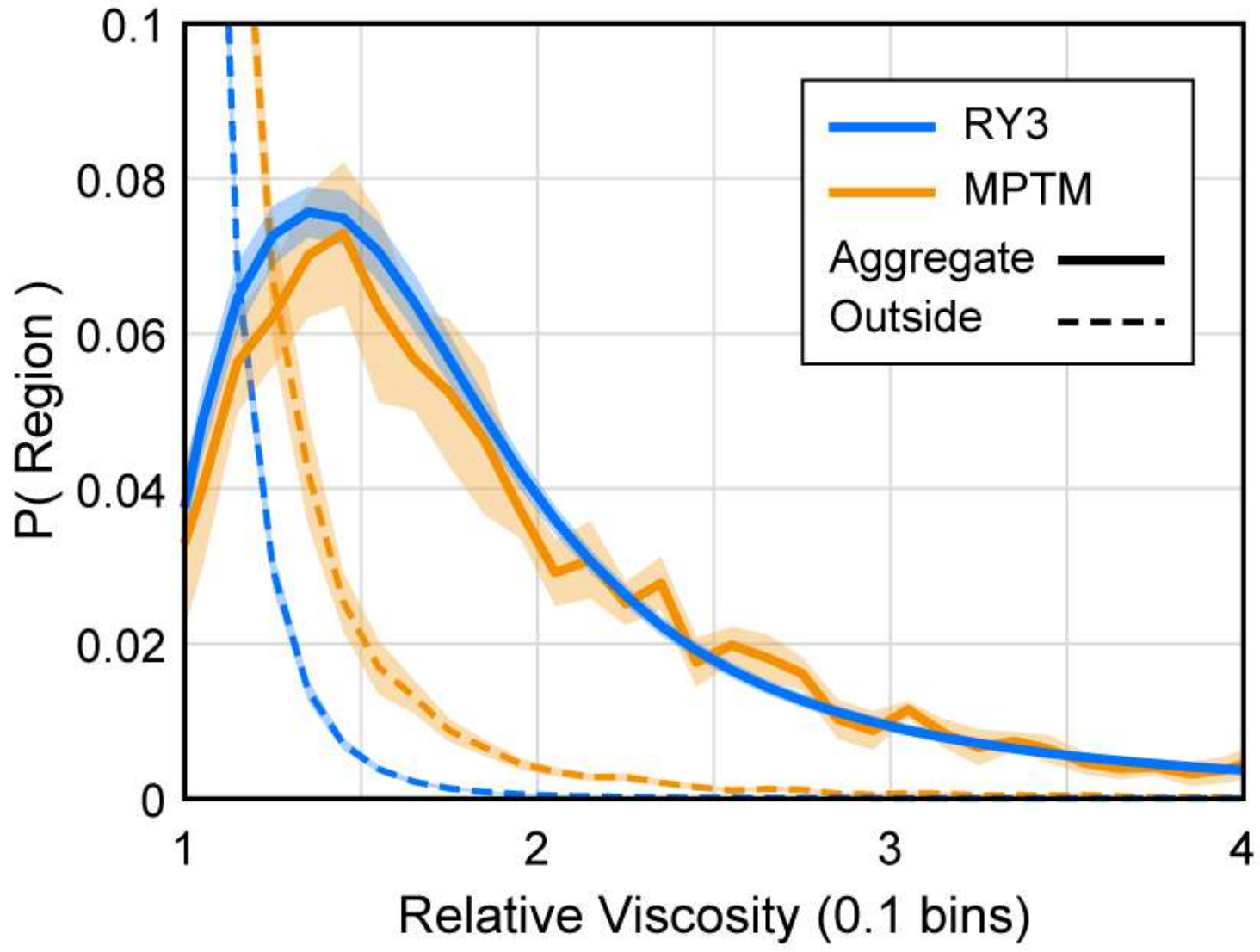

Supplementary Figure 2. **Agreement between relative viscosity probability distributions of diatom aggregates measured with RY3 and MPTM.** The probabilities of relative viscosities from the edge regions (solid lines) or outside (dashed lines, control) of *C. affinis* aggregates, measured with MPTM (orange, Guadayol et al. 2021, N=8 aggregates) and RY3 molecular rotors (blue, this study, N=30 aggregates). The most probable relative viscosity in both MPTM and RY3 outside regions is 1 (seawater). Shaded areas represent the standard error. Source data are provided as a Source Data file.

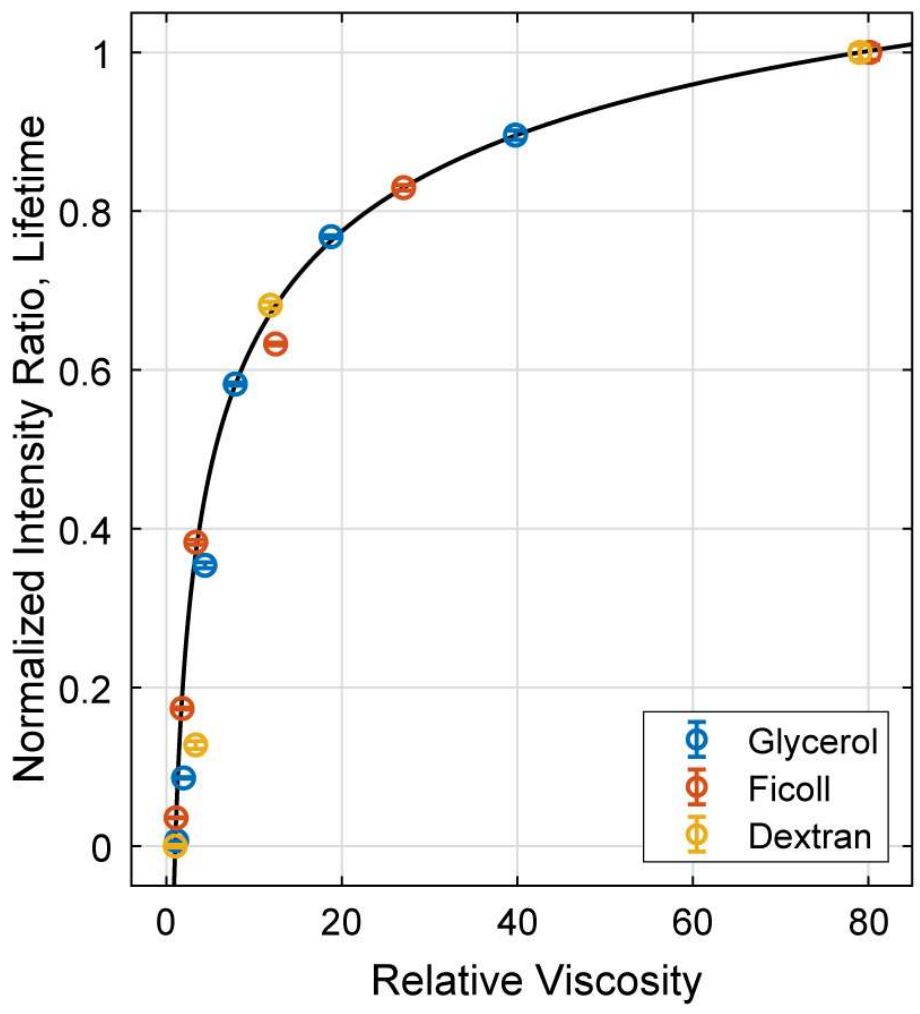

Supplementary Figure 3. **Comparison of calibrations using solutions that vary in rheological complexity**. The RY3 red/blue fluorescence intensity ratio (glycerol, Ficoll) and fluorescence lifetime (dextran) as a function of relative macroviscosity, normalized by their values at the highest common relative viscosity (interpolated between relative viscosity 40 and 275 for glycerol). All three are fit by the power law $x^{-0.2}$ with R^2=0.992 and RMSE=0.037 (black line). Error bars are the standard error with N=3 technical replicates. Source data are provided as a Source Data file.

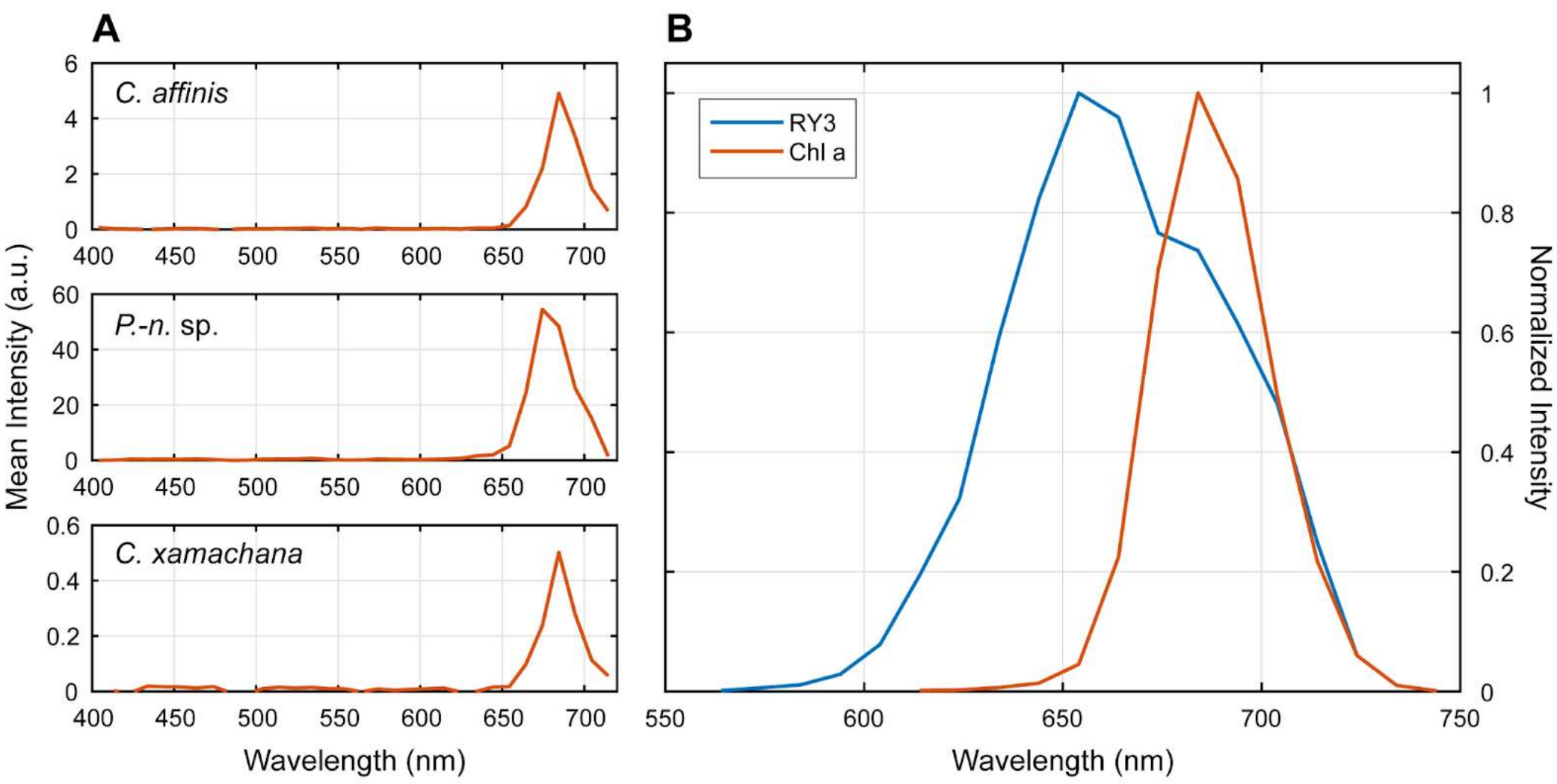

Supplementary Figure 4. Autofluorescence of the the study species. (**A**) Autofluorescence spectra of mucus sampled from *C. affinis*, *P.-n.* sp., and *C. xamachana*. Excitation was 405 and 488 nm, the same as in all experiments. (**B**) The characteristic red RY3 and Chl a spectra used for unmixing experimental images. Source data for **A** and **B** are provided as a Source Data file.

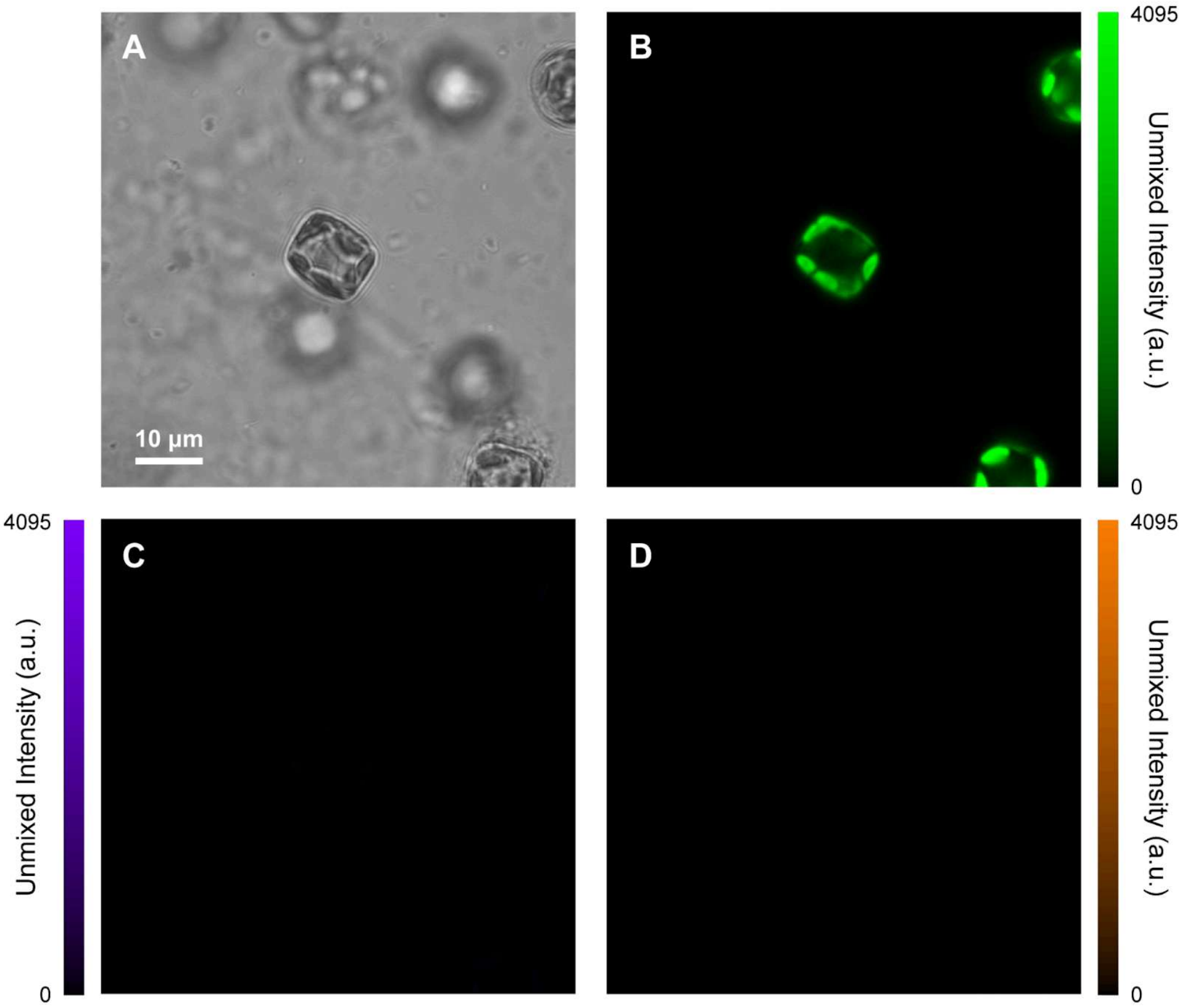

Supplementary Figure 5. Spectral unmixing of Chl a autofluorescence contributes negligible signal to the RY3 channel. A spectrally-unmixed image of *C. affinis* diatom cells with 404 and 488 nm excitation and no dyes. (**A**) Transmitted light showing the locations of the three centric diatom cells. (**B**) The unmixed Chl a channel in green. (**C**) The unmixed RY3 channel in purple. (**D**) The unmixed SYBR Green I channel in orange. Only green is visible, localized to the chloroplasts of the diatom cells. Trace Chl a is present in mucus material located above the diatom on the bottom right.

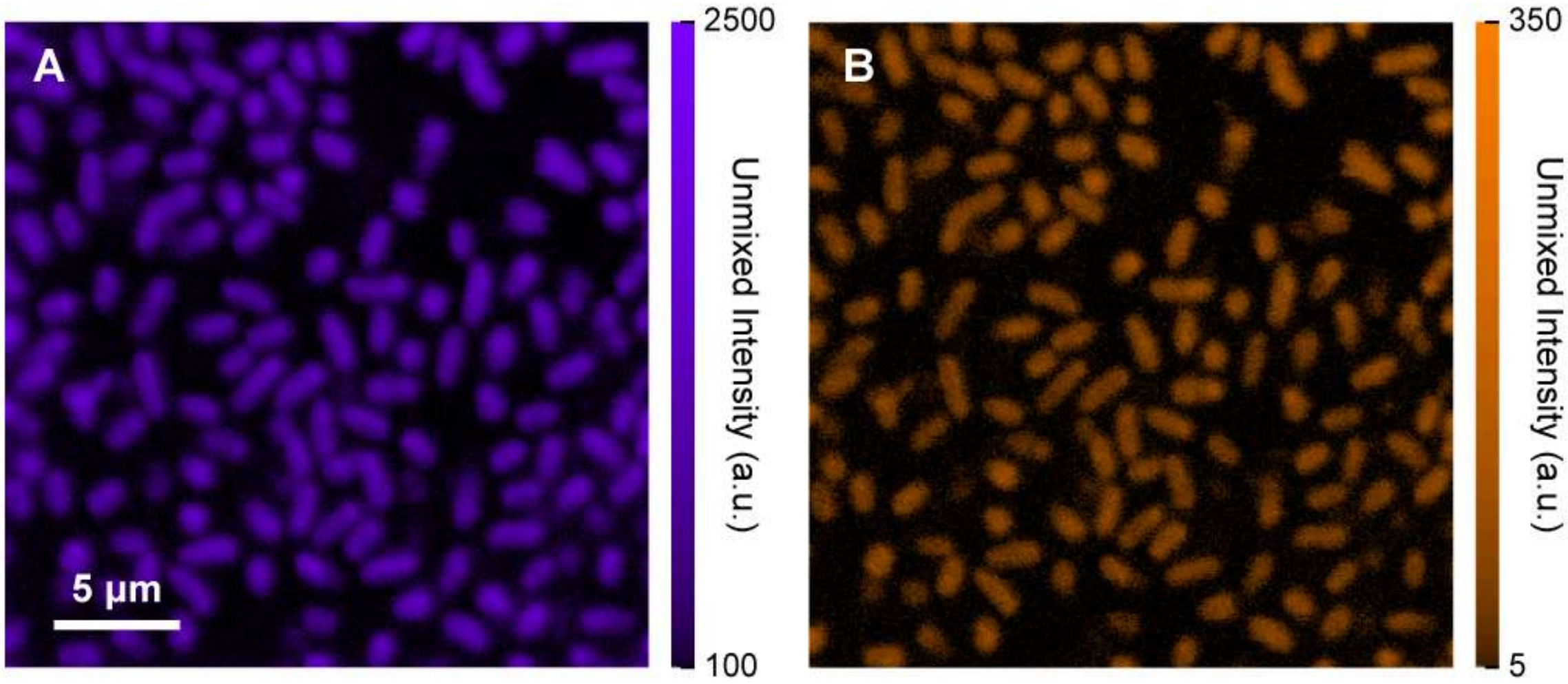

Supplementary Figure 6. **Spatial overlap of bacterial RY3 and SYBR Green I signals.** A confocal microscopy thin section of unmixed RY3 (A) and SYBR Green I (B) fluorescences from a culture of *Pseudoalteromonas* sp. TW7 bacteria.

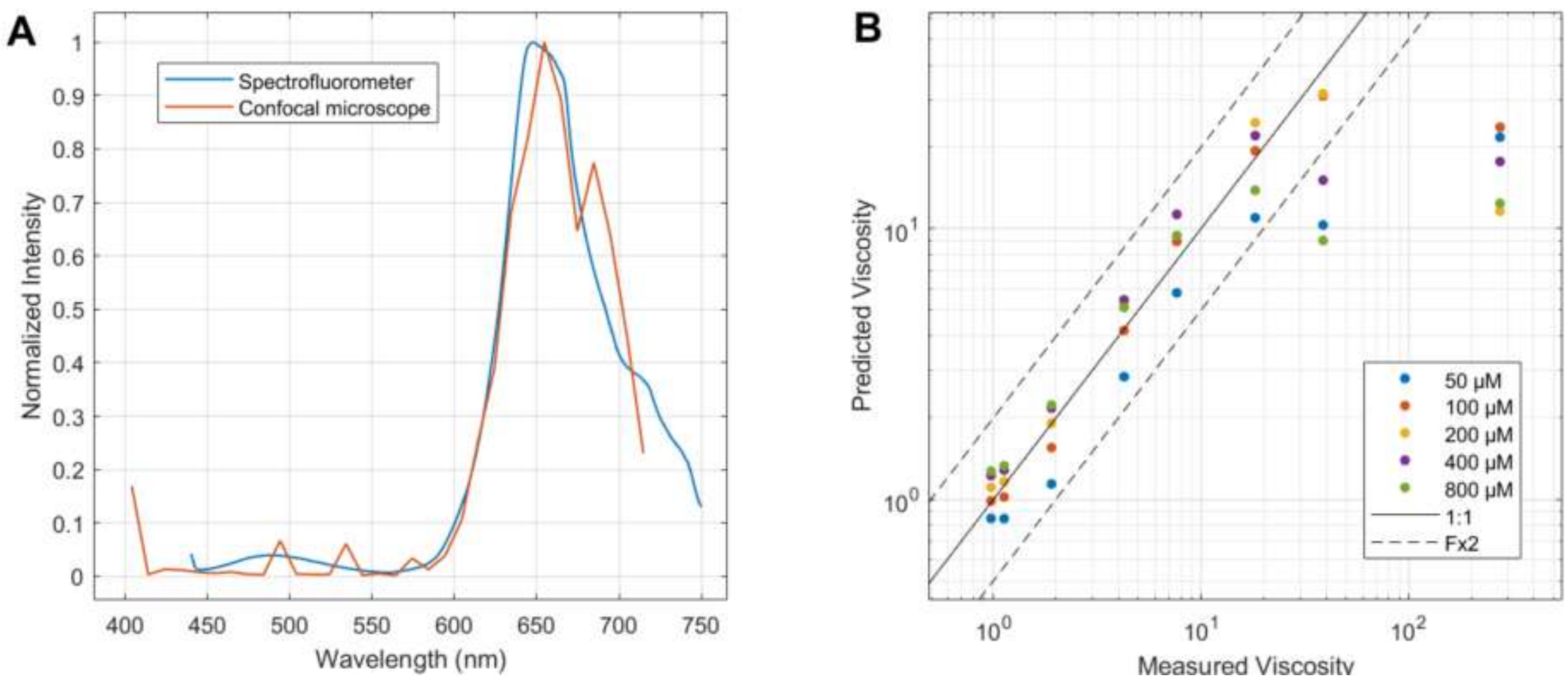

Supplementary Figure 7. **Comparison with the RY3 calibration in Peng et al. 2011.** (A) Comparison of RY3 fluorescence emission spectra measured with a spectrophotometer and a confocal microscope. (B) The performance of the RY3 calibration in Peng et al. 2011 using confocal microscopy fluorescence. Performance is indicated by the closeness of the measured versus predicted relative viscosities to the one-to-one line (solid). Source data for **A** and **B** are provided as a Source Data file.

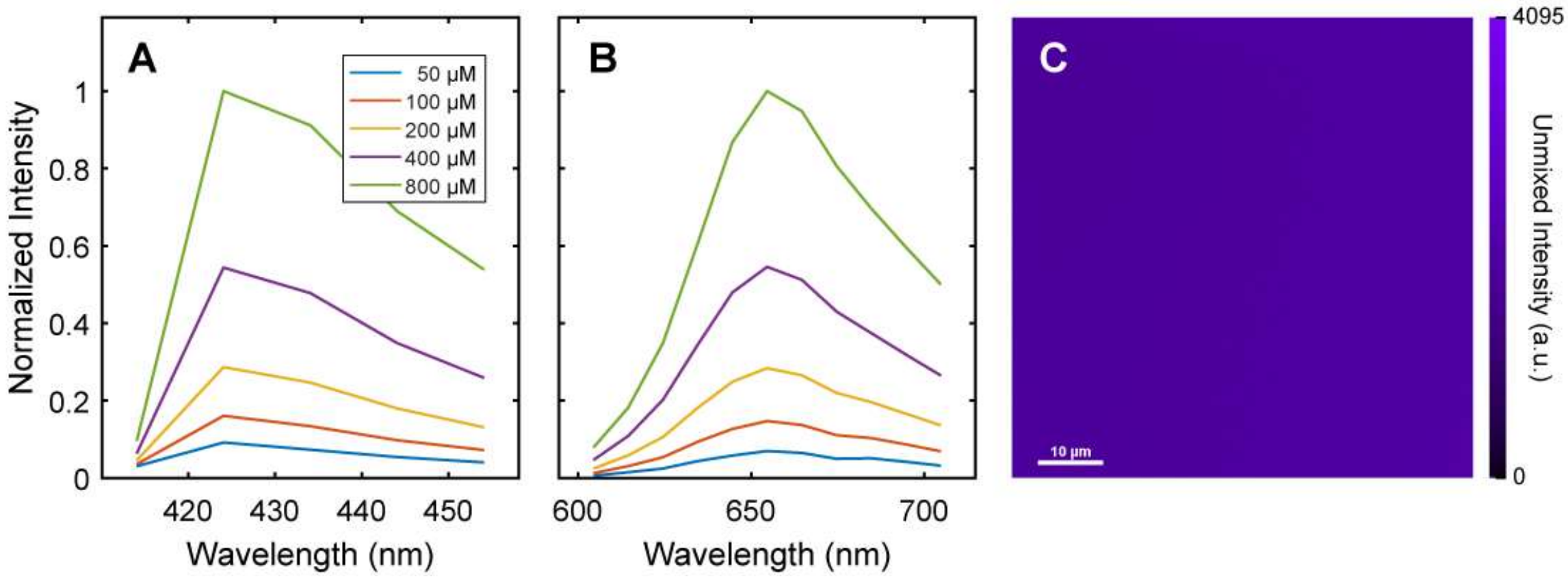

Supplementary Figure 8. **Fluorescence spectra of varying concentrations of RY3 in 18.25 relative viscosity glycerol solutions.** The spectra are normalized by the blue (**A**) or red (**B**) maxima for visualization. (**C**) A confocal microcopy image of unmixed RY3 fluorescence in 800 μM RY3. Source data for **A** and **B** are provided as a Source Data file.

Supplementary Methods

Validation of the molecular rotor method in complex biofluids

Comparison with an independent microrheological technique

Validation of the molecular rotor method in complex biofluids is best demonstrated by agreement with an independent microrheological technique, such as multiple particle tracking microrheology (MPTM). It is not trivial to intercalibrate molecular rotors and MPTM in mucus aggregates: microspheres do not readily penetrate mucus and their Brownian motion interferes with time-averaged RY3 fluorescence imaging. However, we can compare viscosity measurements of mucus aggregates from the same diatom culture (*Chaetoceros affinis*) using MPTM (Guadayol et al. 2020, Supplementary Figure 1C and D) and molecular rotors (our study, Supplementary Figure 1 A and B).

There is strong agreement between the ranges of relative viscosities in aggregates estimated from the two techniques (N=8 in Guadayol et al. 2020; N=30 this study). The pattern of viscosity increasing toward the center of the aggregate is also consistent. Further, the probabilities of relative viscosities in aggregates measured with MPTM corroborate our ratiometric molecular rotor measurements (Supplementary Figure 2).

The MPTM measurements in Guadayol et al. (2021) only represent the outer ~1/3 edges of the aggregates (Supplementary Figure 1E and F), as the authors state "the spatial coverage of the MPTM maps inside the aggregates is limited by the capacity of the microspheres to penetrate the aggregates." For comparison with our molecular rotor measurements, we can select data from edge regions which are defined as pixels from the outer boundary inward 1/3 of the maximum distance inside the aggregate. This definition accommodates complicated aggregate geometry and matches microsphere penetration in Guadayol et al. (2021). The relative viscosity probability distributions of our edge regions show excellent agreement with the MPTM measurements in Guadayol et al. 2020 (Supplementary Figure 2). A two-sided permutation test of Wasserstein distances to determine if the MPTM and RY3 data come from different distributions results in p=0.49 and T_{obs}=-0.051, indicating that the MPTM and RY3 distributions are statistically indistinguishable. This agreement between RY3 and MPTM aggregate viscosity probabilities demonstrates that the molecular rotor viscosity measurements are not significantly influenced by the composition, heterogeneity, or complexity of real mucus material.

Calibration of RY3 in solutions of varying complexity

In addition to glycerol, we calibrated RY3 with Ficoll (400K mw) and dextran (5-40M mw) solutions, both complex fluids with highly-branched polysaccharides (Supplementary Figure 3). We imaged the fluorescence intensity of RY3 in aqueous solutions of glycerol and Ficoll 400 on a Nikon A1R+ confocal microscope. The ratio of red to blue fluorescence of RY3 facilitates comparison of the two viscous agents in a consistent manner. We performed fluorescence lifetime imaging microscopy (FLIM) of aqueous solutions of dextran on a Leica SP8 confocal microscope with a Falcon module. Normalizing the three calibrations by their values at the highest common macroviscosity, they all fall onto a single curve that is fit by a power law (Supplementary Figure 3). The shape of each calibration curve is the same and converting from one to another only requires multiplication by a constant. This demonstrates that the fundamental relationship between RY3 measurements and macroviscosity does not depend on the rheological complexity of the calibration solution.

Spectral unmixing of autofluorescence in mucus samples

The only autofluorescence with 404 and 488 nm excitation in mucus from the diatoms and upside-down jellyfish (zooxanthellae) is consistent with Chl a / PSII with a peak at 685 nm (Supplementary Figure 4A). There is spectral overlap between red RY3 and Chl a emissions, with a 35 nm gap between the two peaks (Supplementary Figure 4B). All imaging was performed with a 32 channel spectral detector (10 nm bins from 400-720nm), providing high spectral resolution for unmixing. The Chl a spectral profile was determined from control samples, and the RY3 spectral profile was determined from viscosity standards. Experimental images were acquired with 4x line averaging to improve the signal-to-noise ratio and spectral unmixing performance. The Chl a and red RY3 profiles were spectrally unmixed from all experimental images with a high degree of accuracy given the spectral resolution, experimentally-determined profiles, consistent optical configuration, and line averaging (Zimmermann 2005; Zimmermann et al. 2014). This procedure accounts for varying contributions of Chl a autofluorescence within and across experimental images on a pixel-by-pixel basis.

To quantify the contribution of Chl a autofluorescence to the unmixed RY3 signal, we can spectrally unmix control images of *C. affinis* diatoms with no dyes and compare the percentage of signal that ends up in the unmixed RY3 channel versus the unmixed Chl a channel (e.g. Supplementary Figure 5). Only 0.44 ± 0.089 % SD of Chl a intensities (N=3, Supplementary Figure 5B) end up in the RY3 channel (Supplementary Figure 5C) after unmixing. In our experimental samples, Chl a is mostly localized to the chloroplasts of diatoms or zooxanthellae (in the case of the jellyfish ephyra). Because we remove these cells in the regions we analyze, there is only a relatively small amount of Chl a present in the analyzed mucus that contributes negligible signal (0.44%) to the unmixed RY3 channel.

Evidence of low RY3 ecotoxicity

No direct assays of the ecotoxicity of RY3 have been made to date. We observed motile bacteria in samples with 50 μM RY3 over 20 hours, which qualitatively indicates low or negligible toxicity towards those bacteria. We counted intact bacterial cells colonizing mucus aggregates in 3D images over time using SYBR Green I and did not detect a significant change in the cell counts for 9 samples over 7 hours or 3 samples over 20 hours (p=0.23 and p=0.095, respectively, in a linear mixed-effects model).

Calibration microscopy

An appropriate optical configuration for the confocal microscope resolves both the weakest and strongest fluorescence signals across all dye and viscosity combinations. In the former case (Fig. 1B), the weak I_B fluorescence for 50 μM RY3 in water must be distinguishable from a blank (i.e. water without dye). Note that water should have a measurable rotor viscosity and not be buried in the noise-floor of the detector. In the latter case (Fig. 1A), the strong I_R fluorescence for 800 μM RY3 in the highest viscosity standard should not saturate the detector. For 4-channel confocal microscopes with filter cubes or adjustable detectors, I_B is best captured at 420-450 nm and I_R at 630-670 nm. Confocal microscopes equipped with 32-channel spectral detectors can capture both signals over the range of 400-720 nm in 10 nm bins. Dichroic mirrors should accommodate 404 nm excitation for RY3, 488 nm excitation for SYBR Green, and 400-700 nm emission. All channels can can be imaged simultaneously. After configuring the detectors and choosing an objective, the main imaging parameters to adjust are laser power, pixel dwell time (scan speed), detector gain, and line averaging. Increasing laser power improves the fluorescence signal but can photobleach dyes (particularly SYBR

Green) and lyse cells. Longer pixel dwell time has a similar effect but also increases imaging time (it is useful for capturing weak fluorescence when laser power has to be at an absolute minimum). Greater gain (detector sensitivity) increases both the fluorescence signal and noise. Line averaging decreases noise and increases imaging time with somewhat less photobleaching than pixel dwell.

Image analysis

Bacteria are identified in the SYBR Green I channel by classifying 3D connected components above a threshold value. The threshold has to be determined manually for each image set because SYBR Green I photobleaches rapidly and produces inconsistent intensity values. A minimum size is also imposed to prevent noise from registering as bacteria. Clusters of bacteria cannot be separated with this method, and so a maximum size is not enforced. There is similar spatial overlap of unmixed RY3 and SYBR Green I signals observed in a culture of *Pseudoalteromonas* sp. TW7 bacteria (Supplementary Figure 6) and bacteria in mucus aggregate images. Therefore, the edges of the bacteria identified with SYBR Green I are dilated outward by 0.4 μm to ensure that any viscosity signal inside the bacterial cells does not overlap with the mucus aggregate viscosities in subsequent analyses. A 0.3 μm-thick 'halo' volume surrounding the spatially-buffered bacterial volume is then defined for calculating the aggregate viscosities with which bacteria are associated.

The boundaries of mucus aggregates are determined using a relative viscosity threshold of 2 and then dilating the edges outward by 0.4-0.8 μm. We tested various edge-detection methods such as spatial viscosity gradients and Sobel and Canny algorithms, but found simple viscosity thresholding and dilation to be the most robust for diffuse aggregate boundaries. The relative viscosity threshold and dilation distance should be adjusted for aggregates from different sources. The viscosity z-stacks are then partitioned into aggregate regions, 'outside' regions, and bacterial halos that overlap with those regions. The probability of a relative viscosity occurring in each of these volumes is calculated to provide quantitative data on the viscous structure of aggregates and bacterial associations.

Comparison with the calibration in Peng et al. 2011

Peng et al. 2011 performed a calibration of 1 μM RY3 in aqueous glycerol solutions using a FP-6500 spectrofluorometer (Jasco, Japan) with 400 nm excitation. When imaging 5 μM RY3 in MCF-7 breast cancer cells using a multiphoton confocal microscope (Leica TCS-SP2) with 800 nm excitation (absorption of two 800 nm photons is equivalent to one 400 nm photon), the authors noted that "In the blue channel (λ_{em} 456 ± 20), however, the fluorescence image is very weak." We also found that RY3 blue fluorescence was weaker in confocal imaging compared to a spectrofluorometer. Supplementary Figure 7A shows the emission spectra of 50 μM RY3 in an aqueous glycerol solution of relative viscosity 4.3, measured with a SpectraMax iD3 spectrofluorometer (Molecular Devices) and a Nikon A1R+ confocal microscope. The spectrofluorometer used broad 400 ± 20 nm excitation that prevented emission measurements below 450 nm, and emission was measured in 1 nm bins. The confocal used narrow 404 nm laser excitation and 10 nm emission bins. The peaks located at 404, 488, and 540 nm are artifacts of the confocal optics. Fluorescence in the 400-500 nm range is an order of magnitude lower when measured with the confocal microscope versus the spectrophotometer.

Although the reason for weaker RY3 blue fluorescence in confocal imaging versus spectrofluorometry is unknown, it is possible that broadband or lower excitation wavelengths in the spectrophotometer yield higher blue fluorescence. Applying the calibration in Peng et al. 2011 to fluorescence measured on a confocal microscope performs worse across all viscosities when compared to the calibration

presented here (Supplementary Figure 7B and Fig. 1E). Relative viscosities greater than 10 cannot be distinguished using this calibration. This highlights the importance of performing molecular rotor calibration on the confocal microscope itself.

High dye concentrations do not influence the photophysics of RY3

At high concentrations, some dyes can self-aggregate and form nanoclusters that alter their photophysics. RY3 fluorescence spectra for the blue and red peaks do not exhibit peak shifts, new peaks, or reduced quantum yields with a 16-fold increase in dye concentration (Supplementary Figure 8A and B). This demonstrates that the photophysics of the rotor are not influenced by high concentrations and that nano-aggregates are not likely present. No aggregates are visible in confocal microscopy images of 800 μM RY3 (e.g. Supplementary Figure 8C).

Experimental determination of the point spread function of the microscope

The point spread function (PSF) of an optical microscope describes the response of the system to a point source of light. Diffraction causes the point source to appear as concentric rings in the x-y plane and an hourglass shape in the x-z plane (Cole et al. 2011). The PSF can be measured experimentally by capturing 3D images of fluorescent spheres that are smaller than the resolution of the microscope. Starting with a 1% solids solution of Fluoro-Max Red 0.1 μm Particles (Thermo Scientific R100TS), dilute by 10^4 in Milli-Q in two steps, vortexing each time. To resolve the full 3D PSF without interference from the glass coverslip, 25 uL of the diluted fluorescent spheres can be mixed with 975 uL of an aqueous 25% Pluronic F-127 solution (Sigma-Aldrich P2443) at 4 °C and then pipetted into a chambered coverglass slide for imaging. The Pluronic solution transitions from a liquid at 4 °C to a solid at higher temperatures, fixing the particles in place as the confocal laser heats the solution during imaging. The refractive index of an aqueous Pluronic solution (n=1.36, Kim et al., 2018) is similar to seawater (n=1.34), making it ideal for imaging fluorescent spheres away from the coverglass. Z-stacks of fluorescent spheres are imaged with 0.02 μm horizontal and 0.1 μm vertical pixel resolution, half moving from bottom to top and half from top to bottom to account for photobleaching. The z-stacks are then aligned using cross sections of the fluorescence intensity and averaged to obtain the final PSF. The horizontal and vertical full width at half maximum intensities of the PSF provide the true resolution of the microscope.

Cultures and animals

The *Chaetoceros affinis* Lauder diatom (Bigelow CCMP159) was cultured on an orbital shaker with f/2 media in filtered seawater at 22 °C and a 14:10 hour light:dark cycle. The *Pseudo-nitzschia* sp. diatom was isolated from the pier at Scripps Institution of Oceanography and cultured at 18 °C with a 12:12 hour light:dark cycle. The species was not identified via 18s or ITS amplicon sequencing. One week old ephyrae of the upside-down jellyfish *Cassiopea xamachana* were kept in 6-well culture plates at 25 °C and 35 ppt seawater with 12:12 hour light:dark cycle. Ephyrae were fed *Artemia* sp. brine shrimp every other day. Any uneaten brine shrimp were removed after feeding by exchanging 50% of the seawater.

Supplementary Discussion

FLIM vs. ratiometry

There are two main options for concentration-independent fluorescence measurements: ratiometric (dual-emission) fluorophores or fluorescence lifetime imaging microscopy (FLIM). Ratiometric molecular rotors fluoresce at two different wavelengths, one that is more sensitive to viscosity and the other that can be used as a reference for the concentration of the dye. Dividing the viscosity signal by the reference signal yields a concentration-independent ratio of intensities that can be calibrated to viscosity. The drawbacks of using ratiometric fluorophores are that measuring two emissions reduces the spectral bandwidth remaining for other probes, and taking the ratio of two image channels can amplify noise and generate divide-by-zero errors. FLIM records the fluorescence lifetime of a molecular rotor as opposed to the fluorescence intensity. After an excitation pulse, the decay of rotor fluorescence over time varies with viscosity but not dye concentration. FLIM often takes much longer than standard confocal imaging, which can further stress living organisms. FLIM-equipped confocal microscopes utilize pulsed lasers and specialized hardware that are more expensive and less common than standard confocal microscopes, and fitting a model to a fluorescence decay curve to produce a lifetime is not always straightforward. Further, separating autofluorescence from the molecular rotor signal is difficult if not impossible with FLIM. Both fluorescence ratiometry and FLIM have the added benefit of accounting for variability in laser intensity, sample bleaching, and other optical artifacts during imaging.

Hydrophilic molecular rotors

Imaging the viscosity of mucus material and associated bacteria imposes the following requirements for a molecular rotor: The molecule must be (1) hydrophilic and (2) fairly small to readily penetrate into aqueous mucus material. (3) The rotor should exhibit low toxicity to microbes and other cells of interest. (4) The fluorescence excitation and emission wavelengths should both lie in the visible spectrum for use with standard confocal microscopes; furthermore, ultraviolet light can be harmful to both microbes and microscopists. (5) The molecular rotor must be capable of producing a measurement of viscosity that is independent of the concentration of the dye via ratiometric fluorescence or FLIM, because the dye may not be uniformly distributed at the microscale in a complex biofluid.

The best-characterized hydrophilic molecular rotor that is commercially available is 9-(2-carboxy-2-cyanovinyl) julolidine (CCVJ). Unfortunately, CCVJ is not a ratiometric fluorophore and it exhibits picosecond lifetimes that are too short to be resolved by most modern FLIM systems (Gavvala et al. 2015). Normal laboratory lighting conditions also cause CCVJ to rapidly convert into a non-fluorescent isomer, complicating its usage in microscopy (Rumble et al. 2012). Thioflavin T can be used as a molecular rotor, but it is non-ratiometric, highly toxic, and binds strongly to proteins (Thompson et al. 2015). 4-DASPI is a non-ratiometric and hydrophilic molecular rotor with a high affinity for surfaces and picosecond lifetimes (Mirzahossein et al. 2022; Lyons et al. 2023). Sulfo-BODIPY is a molecular rotor with nanosecond lifetimes that localizes to aqueous plant cell vacuoles and may be useful for imaging mucus (Michels et al. 2020). A coumarin-BODIPY molecular rotor exhibits both ratiometric fluorescence and nanosecond lifetimes, but has full spectral overlap with SYBR Green I (Yang et al. 2013). Cy3 is a non-ratiometric and hydrophilic molecular rotor that is less sensitive to lower viscosities than RY3, but is commercially available and can be paired with

Cy5 (which has a similar structure but is not a molecular rotor) for a pseudo-ratiometric measurement (Luby-Phelps et al. 1993; Hosny et al. 2016).

Supplementary References

Cole, R. W., Jinadasa, T., & Brown, C. M. (2011). Measuring and interpreting point spread functions to determine confocal microscope resolution and ensure quality control. *Nature Protocols*, *6*(12), 1929–1941. https://doi.org/10.1038/nprot.2011.407

Gavvala, K., Satpathi, S., & Hazra, P. (2015). Ultrafast dynamics of a molecular rotor in chemical and biological nano-cavities. *RSC Advances*, *5*(89), 72793–72800. https://doi.org/10.1039/C5RA13298C

Guadayol, Ò., Mendonca, T., Segura-Noguera, M., Wright, A. J., Tassieri, M., & Humphries, S. (2021). Microrheology reveals microscale viscosity gradients in planktonic systems. *Proceedings of the National Academy of Sciences*, *118*(1), e2011389118. https://doi.org/10.1073/pnas.2011389118

Guadayol, Ò., Segura-Noguera, M., & Humphries, S. (2020). A collection of 2-D viscosity maps around phytoplankton cells and aggregates. figshare. Collection. https://doi.org/10.6084/m9.figshare.c.4534553.v3

Hosny, N. A., Fitzgerald, C., Vyšniauskas, A., Athanasiadis, A., Berkemeier, T., Uygur, N., Pöschl, U., Shiraiwa, M., Kalberer, M., Pope, F. D., & Kuimova, M. K. (2016). Direct imaging of changes in aerosol particle viscosity upon hydration and chemical aging. *Chemical Science*, *7*(2), 1357–1367. https://doi.org/10.1039/C5SC02959G

Kim, M., Vala, M., Ertsgaard, C. T., Oh, S.-H., Lodge, T. P., Bates, F. S., & Hackel, B. J. (2018). Surface Plasmon Resonance Study of the Binding of PEO–PPO–PEO Triblock Copolymer and PEO Homopolymer to Supported Lipid Bilayers. *Langmuir*, *34*(23), 6703–6712. https://doi.org/10.1021/acs.langmuir.8b00873

Luby-Phelps, K., Mujumdar, S., Mujumdar, R. B., Ernst, L. A., Galbraith, W., & Waggoner, A. S. (1993). A novel fluorescence ratiometric method confirms the low solvent viscosity of the cytoplasm. *Biophysical Journal*, *65*(1), 236–242. https://doi.org/10.1016/S0006-3495(93)81075-0

Lyons, A., Zickus, V., Álvarez-Mendoza, R., Triggiani, D., Tamma, V., Westerberg, N., Tassieri, M., & Faccio, D. (2023). Fluorescence lifetime Hong-Ou-Mandel sensing. *Nature Communications*, *14*(1), 8005. https://doi.org/10.1038/s41467-023-43868-x

Michels, L., Gorelova, V., Harnvanichvech, Y., Borst, J. W., Albada, B., Weijers, D., & Sprakel, J. (2020). Complete microviscosity maps of living plant cells and tissues with a toolbox of targeting mechanoprobes. *Proceedings of the National Academy of Sciences*, *117*(30), 18110–18118. https://doi.org/10.1073/pnas.1921374117

Mirzahossein, E., Grzelka, M., Guerton, F., Bonn, D., & Brown, R. (2022). Adsorption of a water-soluble molecular rotor fluorescent probe on hydrophobic surfaces. *Scientific Reports*, *12*(1), 22197. https://doi.org/10.1038/s41598-022-26722-w

Peng, X., Yang, Z., Wang, J., Fan, J., He, Y., Song, F., Wang, B., Sun, S., Qu, J., Qi, J., & Yan, M. (2011). Fluorescence Ratiometry and Fluorescence Lifetime Imaging: Using a Single Molecular Sensor for Dual Mode Imaging of Cellular Viscosity. *Journal of the American Chemical Society*, *133*(17), 6626–6635. https://doi.org/10.1021/ja1104014

Rumble, C., Rich, K., He, G., & Maroncelli, M. (2012). CCVJ Is Not a Simple Rotor Probe. *The Journal of Physical Chemistry A*, *116*(44), 10786–10792. https://doi.org/10.1021/jp309019g

Thompson, A. J., Herling, T. W., Kubánková, M., Vyšniauskas, A., Knowles, T. P. J., & Kuimova, M. K. (2015). Molecular Rotors Provide Insights into Microscopic Structural Changes During Protein Aggregation. *The Journal of Physical Chemistry B*, *119*(32), 10170–10179. https://doi.org/10.1021/acs.jpcb.5b05099

Yang, Z., He, Y., Lee, J.-H., Park, N., Suh, M., Chae, W.-S., Cao, J., Peng, X., Jung, H., Kang, C., & Kim, J. S. (2013). A Self-Calibrating Bipartite Viscosity Sensor for Mitochondria. *Journal of the American Chemical Society*, *135*(24), 9181–9185. https://doi.org/10.1021/ja403851p

Zimmermann, T. (2005). Spectral Imaging and Linear Unmixing in Light Microscopy. In J. Rietdorf (Ed.), *Microscopy Techniques: -/-* (pp. 245–265). Springer. https://doi.org/10.1007/b102216

Zimmermann, T., Marrison, J., Hogg, K., & O'Toole, P. (2014). Clearing Up the Signal: Spectral Imaging and Linear Unmixing in Fluorescence Microscopy. In S. W. Paddock (Ed.), *Confocal Microscopy: Methods and Protocols* (pp. 129–148). Springer. https://doi.org/10.1007/978-1-60761-847-8_5
