# Supplementary material for: Molecular rotors reveal the 3D viscous habitat of mucus-colonizing bacteria
Source: Nat Commun. 2026 Jul 22;17:8952. doi: 10.1038/s41467-026-75892-y (PMC13503797; doi:10.1038/s41467-026-75892-y)
Supplement: Supplementary file 2 [file 41467_2026_75892_MOESM2_ESM.pdf]

Description of Additional Supplementary Files

File Name: Supplementary Movie 1

Description: Bacteria (orange) colonize a ~50 μm-thick mucus layer (blue) surrounding a *C. xamachana* ephyra (green).
