# Supplementary material for: Molecular rotors reveal the 3D viscous habitat of mucus-colonizing bacteria
Source: Nat Commun. 2026 Jul 22;17:8952. doi: 10.1038/s41467-026-75892-y (PMC13503797; doi:10.1038/s41467-026-75892-y)
Supplement: Supplementary file 3 [file 41467_2026_75892_MOESM4_ESM.pdf]

nature portfolio

Corresponding author(s): B.G. Inman

Last updated by author(s): Jun 23, 2026

Reporting Summary

Nature Portfolio wishes to improve the reproducibility of the work that we publish. This form provides structure for consistency and transparency in reporting. For further information on Nature Portfolio policies, see our Editorial Policies and the Editorial Policy Checklist.

Statistics

For all statistical analyses, confirm that the following items are present in the figure legend, table legend, main text, or Methods section.

n/a	Confirmed	
☐	☒	The exact sample size (*n*) for each experimental group/condition, given as a discrete number and unit of measurement
☐	☒	A statement on whether measurements were taken from distinct samples or whether the same sample was measured repeatedly
☐	☒	The statistical test(s) used AND whether they are one- or two-sided<br>*Only common tests should be described solely by name; describe more complex techniques in the Methods section.*
☒	☐	A description of all covariates tested
☐	☒	A description of any assumptions or corrections, such as tests of normality and adjustment for multiple comparisons
☐	☒	A full description of the statistical parameters including central tendency (e.g. means) or other basic estimates (e.g. regression coefficient) AND variation (e.g. standard deviation) or associated estimates of uncertainty (e.g. confidence intervals)
☐	☒	For null hypothesis testing, the test statistic (e.g. *F*, *t*, *r*) with confidence intervals, effect sizes, degrees of freedom and *P* value noted<br>*Give P values as exact values whenever suitable.*
☒	☐	For Bayesian analysis, information on the choice of priors and Markov chain Monte Carlo settings
☒	☐	For hierarchical and complex designs, identification of the appropriate level for tests and full reporting of outcomes
☒	☐	Estimates of effect sizes (e.g. Cohen's *d*, Pearson's *r*), indicating how they were calculated

Our web collection on statistics for biologists contains articles on many of the points above.

Software and code

Policy information about availability of computer code

Data collection	NIS-Elements AR v5.21.02, TA Instruments TRIOS v5.11.0.31
Data analysis	MATLAB R2024b, MATLAB Image Processing Toolbox v24.2, MATLAB Curve Fitting Toolbox v24.2, Bio-Formats MATLAB Toolbox v8.3.0, MATLAB bwdistsc v.1.8.0, and MATLAB ry3_analysis custom code provided in a Github repository with a Zenodo DOI.

For manuscripts utilizing custom algorithms or software that are central to the research but not yet described in published literature, software must be made available to editors and reviewers. We strongly encourage code deposition in a community repository (e.g. GitHub). See the Nature Portfolio guidelines for submitting code & software for further information.

Data

Policy information about availability of data

All manuscripts must include a data availability statement. This statement should provide the following information, where applicable:

- Accession codes, unique identifiers, or web links for publicly available datasets
- A description of any restrictions on data availability
- For clinical datasets or third party data, please ensure that the statement adheres to our policy

The microscopy data generated in this study have been deposited in Dryad with the DOI 10.5061/dryad.x3ffbg818 [https://doi.org/10.5061/dryad.x3ffbg818]. Unless otherwise stated, all data supporting the results of this study can be found in the article, supplementary, and source data files. Source data are provided with this paper.

Research involving human participants, their data, or biological material

Policy information about studies with human participants or human data. See also policy information about sex, gender (identity/presentation), and sexual orientation and race, ethnicity and racism.

Reporting on sex and gender	This study does not involve humans.
Reporting on race, ethnicity, or other socially relevant groupings	This study does not involve humans.
Population characteristics	This study does not involve humans.
Recruitment	This study does not involve humans.
Ethics oversight	This study does not involve humans.

Note that full information on the approval of the study protocol must also be provided in the manuscript.

Field-specific reporting

Please select the one below that is the best fit for your research. If you are not sure, read the appropriate sections before making your selection.

☒ Life sciences ☐ Behavioural & social sciences ☐ Ecological, evolutionary & environmental sciences

For a reference copy of the document with all sections, see nature.com/documents/nr-reporting-summary-flat.pdf

Life sciences study design

All studies must disclose on these points even when the disclosure is negative.

Sample size	This concept paper focuses on individual mucus aggregates (N=1 each) from three sources (Chaetoceros affinis diatom culture, Pseudo-nitzschia sp. diatom culture, Cassiopea xamachana jellyfish) to showcase the utility of the molecular rotor method for extracting quantitative data from individual samples. Within a sample, the relevant statistical quantity is the number of imaging voxels in which the viscosity was calculated, which were 28311552, 44040192, and 42991616 voxels, respectively. The data is presented as viscosity probability distributions. Because the probability distributions are highly skewed, statistical parameters such as the mean, median, and standard deviation are not as informative as the probability distributions themselves.
Data exclusions	To analyze single mucus aggregates, any other partial mucus aggregates that encroached on the imaging volume were removed from the analysis. This was a pre-established exclusion criteria.
Replication	Three separate examples are used in the study to showcase the molecular rotor method. No replicates were included for each example because the study investigates differences between individual particles instead of population level effects.
Randomization	Randomization was not relevant to this study because organisms were not allocated into experimental groups.
Blinding	Blinding was not relevant to this study because organisms were not allocated into experimental groups.

Reporting for specific materials, systems and methods

We require information from authors about some types of materials, experimental systems and methods used in many studies. Here, indicate whether each material, system or method listed is relevant to your study. If you are not sure if a list item applies to your research, read the appropriate section before selecting a response.

Materials & experimental systems

n/a	Involved in the study	
☒	☐	Antibodies
☐	☒	Eukaryotic cell lines
☒	☐	Palaeontology and archaeology
☐	☒	Animals and other organisms
☒	☐	Clinical data
☒	☐	Dual use research of concern
☒	☐	Plants

Methods

n/a	Involved in the study	
☒	☐	ChIP-seq
☒	☐	Flow cytometry
☒	☐	MRI-based neuroimaging

Eukaryotic cell lines

Policy information about cell lines and Sex and Gender in Research

Cell line source(s)	Chaetoceros affinis diatoms: Bigelow CCMP159. Pseudo-nitzschia sp. diatoms: isolated from the pier at Scripps Institution of Oceanography, and was not identified via 18s or ITS amplicon sequencing. The sex of diatom cells can not be determined.
Authentication	Not authenticated, because this is not a standard procedure for diatoms.
Mycoplasma contamination	Not tested for mycoplasma contamination, because this is not a standard procedure for diatoms.
Commonly misidentified lines (See ICLAC register)	None of the diatoms used in this study are included in the ICLAC register of misidentified cell lines.

Animals and other research organisms

Policy information about studies involving animals; ARRIVE guidelines recommended for reporting animal research, and Sex and Gender in Research

Laboratory animals	The study utilized one-week old ephyrae of the upside-down jellyfish Cassiopea xamachana.
Wild animals	The study did not use wild animals.
Reporting on sex	Jellyfish ephyrae do not exhibit known sexual dimorphism.
Field-collected samples	The ephyrae were kept in 6-well culture plates at 25 °C and 35 ppt seawater with 12:12 hour light:dark cycle. Ephyrae were fed Artemia sp. brine shrimp every other day. Any uneaten brine shrimp were removed after feeding by exchanging 50% of the seawater.
Ethics oversight	Use of Cassiopea xamachana jellyfish ephyrae in experimental studies does not currently require ethical approval or permitting, as per the United States Animal Welfare Act USC 2132(g) and the Institutional Animal Care and Use Committee. We endeavored to minimize harm of the jellyfish and adhere to ethical best practices by performing experiments on a minimal number of whole organisms.

Note that full information on the approval of the study protocol must also be provided in the manuscript.

Plants

Seed stocks	This section was marked n/a above, but still appeared in the PDF.
Novel plant genotypes	This section was marked n/a above, but still appeared in the PDF.
Authentication	This section was marked n/a above, but still appeared in the PDF.
