# Supplementary material for: Molecular rotors reveal the 3D viscous habitat of mucus-colonizing bacteria
Source: Nat Commun. 2026 Jul 22;17:8952. doi: 10.1038/s41467-026-75892-y (PMC13503797; doi:10.1038/s41467-026-75892-y)
Supplement: Supplementary file 4 [file 41467_2026_75892_MOESM5_ESM.pdf]

nature portfolio

Peer Review File

Molecular rotors reveal the 3D viscous habitat of mucus-colonizing bacteria

Corresponding Author: Dr Bryce Inman

This file contains all reviewer reports in order by version, followed by all author rebuttals in order by version.

Version 1:

Reviewer comments:

Reviewer #1

(Remarks to the Author)
In this paper, Inman et al, have described a method to quantify and map viscosity of 'undisturbed mucus and associated bacteria' using a viscosity-sensing fluorescent ratiometric probe, RY3. Further the authors have applied their method in mucosa produced by two diatom species as well as mucosa surrounding upside down jelly fish. Further authors have attempted to link viscosity of the mucosa to specific aspects of the organisms. Authors state that "Individual mucus aggregates from cultures of Chaetoceros affinis and Pseudo-nitzschia sp. diatoms exhibit consolidated versus patchy viscous morphologies, respectively, that result in distinct patterns of microbial colonization." Authors also find that mucus surrounding upside down jellyfish Cassiopea xamachana to be viscous such that a microbiome is maintained while minimizing microbial contact. For their work, authors examine the blue and red emission from the previously described molecular rotor dye RV3 and describe a ratiometric readout. It is to be noted that the raw ratiometric readout is not suitable due to confounding factors like autofluorescence and hence they attempt to develop a calibration curve in-vitro using water-glycerol mixtures and then extending this technique to mucus in three distinct biological contexts.
While the paper presents an interesting and welcome application of a micro-environment sensitive fluorescence probe to 'undisturbed' mucosa surrounding distinct biological entities, several open questions remain. Specifically there appear to be important caveats and potential limitations of the fluorescence probing/ratiometric approach that are not addressed in the opinion of this reviewer. Therefore it is not clear that the readout being studied is faithfully reporting the viscosity of the mucus. It is plausible the readout is being convoluted by other complicating factors like polarity, differential autofluorescence and chemical composition of the media as well differential scattering from the 3-D bioaggregates. Further, in the absence of supporting experiments through independent methods and specific validation of the probe in complex, microheterogeneous media, the data presented in this paper may not support conclusions presented here. Specifically the study notes correlations of bacterial penetration and colonisations with differential viscosity readouts as described here. It is not clear whether these are incidental correlations or if there is a causal relationship between 'viscosity' and any aspects of the complex mucosa.

Specific points:
1. Differential and changing contributions from mucosa autofluorescence to the red and blue channels would significantly compromise the reporting of viscosity from the dye, using the method as described here. This is particularly relevant when comparing viscosity in two distinct biological milieu, wherein the contribution of autofluorescence may be very different.
2. An important finding of the paper as described here is the comparison of viscosity from two different mucosa surrounding distinct diatom species. Clearly the chemical composition would be different. It is not clear from this study if confounding factors like specific chemical interactions, polarity and factors other than viscosity would differentially effect the blue and red channels. This needs to be addressed in detail.
3. Authors compare FLIM and ratiometric approaches. Authors have claimed that "it is difficult or impossible to separate overlapping autofluorescence with FLIM". They have referred to the Supplementary information section to justify their claim but surprisingly no quantifiable arguments have been provided. What was the contribution from AF of Chlorophyll and related pigments in the red emission region of RY3? This is an important factor that is not fully addressed. In general AF have lower lifetime and needs high laser power to image and therefore can easily be separated from dye lifetime either by using low laser power or by careful analysis using gating. Chlorophyll can have a range of lifetime depending on whether its in free or bound form. It would have been instructive to see the AF distribution in the used experimental setting.
4. The authors use exclusively use RY3 for the purpose of quantifying viscosity. Rationale is not clear. For instance There are ratiometric and FLIM dyes (for instance see Yang et al, JACS 2013) for viscosity with emissions at blue and green region

that could have been used to avoid AF at far red region if there was a huge contribution from AF. In that context some complex analysis and calibration steps mentioned in the paper could have been avoided.
5. RY3 is a lipophilic cationic dye and also cell permeable. No experiment has been conducted to check if RY3 could stain the colonizing bacteria. It is possible that signal that is presumed to be from mucosa alone is indeed from the bacterial membranes as well. A simple control experiment using any bacteria could have been performed to address this.
6. The concentration of RY3 used for this study is very high compared to the original paper. Such amphipathic dyes are known to self-aggregate and form nanocluster where its photophysics might change. It would have been nice to provide fluorescence spectra for the dye concentrations mentioned at Fig. 1A. Additionally, a control experiment of imaging the dye in solution can also give information about self-aggregation induced formation of nano-aggregates.
7. The whole technique rests on a calibration curve that is developed in solution. This complex calibration curve is then used in highly micro-heterogeneous mucus and a comparison is made between putative viscosity readouts from distinct mucus samples originating from distinct bio-specimens/species. Directly relating calibration curves obtained in solution/solvents to highly complex and varied media is fraught with risk. Validation of the calibration curves in controlled samples (amenable to systematic modulation of viscosity) and that resemble the mucus would need to be performed.
8. A significant aspect of this study is the comparison of the viscosities from the two diatom mucosa and how this may explain differential bacterial penetration and colonizing. Are there other explanations of differential penetration and colonizing. What is the evidence that viscosity is the causal factor? Can such correlations arise due to other factors?
9. It is not clear how scattering from mucosa may differentially affect the blue and red channels and how this may change depending on the context. This may significantly compromise the calibration used here.
10. Minor point: Further, this reviewer can see black area between the orange circle and SYBR 1 signal (DNA) in Fig. 2 and Fig. 4. No explanation has been offered for this.
Overall while this is an interesting attempt, there appears to be significant caveats that are not fully addressed here. Also this is a study that presents potentially interesting correlations but it’s not clear how it may be possible to definitively link viscosity as the key factor that governs specific features of mucosa especially when comparing diverse samples.

(Remarks on code availability)

Reviewer #2

(Remarks to the Author)
I co-reviewed this manuscript with one of the reviewers who provided the listed reports. This is part of the Nature Communications initiative to facilitate training in peer review and to provide appropriate recognition for Early Career Researchers who co-review manuscripts.

(Remarks on code availability)

Reviewer #3

(Remarks to the Author)
This is an intriguing manuscript that describes the locations of bacteria and phytoplankton in three contexts. The use of the molecular rotor RY3 seems novel and some insights about the distribution of these microorganisms has been provided. I have some concerns that need addressing to be more certain about the manuscript's conclusions.

Major comments:

How many times was the calibration presented in figure 1 repeated? If only once what is the justification.

Why was only 1 aggregation analyzed for each diatom? I understand that many microorganisms were looked at within the aggregation, but I am perplexed that 1 aggregation is enough to rule out chance playing an outsized role here.

It seems that only 1 Cassiopea ephyra was analyzed in figure 4. Is this correct? If so, why not improve the rigor with some reproduction of the results?

Minor comments:

It is worthwhile to describe marine snow directly in the introduction. It may be useful for a more general audience.

Should the title be more inclusive of diatoms?

(Remarks on code availability)

Version 2:

Reviewer comments:

Reviewer #1

(Remarks to the Author)
The authors have addressed many of the questions and concerns raised earlier.

We feel the following points may be addressed through textual changes:

1) The authors note that the observations linking microviscoslity changes to bacterial colonisation are correlative and no casual relationship can be inferred. They have appropriately added a section in the discussion.
However in the abstract they state :
" Individual examples of mucus aggregates from cultures of Chaetoceros affinis and Pseudo-nitzschia sp. diatoms exhibit consolidated versus patchy viscous morphologies, respectively, that result in distinct patterns of microbial colonization."

This implies causality, in fact explicitly stating that 'distinct patterns ..of microbial colonisation' result from patchy viscous morphologies. This needs to be toned down, wherein the finding of patchy morphologies needs to be described ALONG WITH specific patterns of microbial colonisation

2) The authors have presented detailed arguments on methodology in the response. These reviewers feel it would be beneficial to include a short section in the discussion where the caveats and assumptions underlying. the methodology used may be articulated. This section may also mention other potential ways to address viscosity (like FLIM) and these methods may be briefly compared. FLIM for instance, is independent of intensity fluctuations (even potentially autofluorescence contributions through lifetime gating) but requires specialised equipment and so on.

(Remarks on code availability)

Reviewer #2

(Remarks to the Author)
I co-reviewed this manuscript with one of the reviewers who provided the listed reports. This is part of the Nature Communications initiative to facilitate training in peer review and to provide appropriate recognition for Early Career Researchers who co-review manuscripts.

(Remarks on code availability)

Reviewer #3

(Remarks to the Author)
The authors have taken some steps to tone down the claims in the paper based on its level of rigor. The method used is innovative and is likely to reveal much. Overall the manuscript is improved, but since the method is the key aspect the authors want to get across to the reader. They need to continue to be careful about the biological information provided by these studies given their low level of replication.

For example:
“A viscous mucus layer surrounding an ephyra of the upside-down jellyfish Cassiopea xamachana maintains a local microbiome while preventing direct contact with the animal.” In the abstract should be:
“A viscous mucus layer surrounding an ephyra of the upside-down jellyfish Cassiopea xamachana may maintain a local microbiome while preventing direct contact with the animal.”

I appreciate the authors efforts at improving the manuscript.

(Remarks on code availability)

Open Access This Peer Review File is licensed under a Creative Commons Attribution 4.0 International License, which permits use, sharing, adaptation, distribution and reproduction in any medium or format, as long as you give appropriate credit to the original author(s) and the source, provide a link to the Creative Commons license, and indicate if changes were made.
In cases where reviewers are anonymous, credit should be given to 'Anonymous Referee' and the source.
The images or other third party material in this Peer Review File are included in the article's Creative Commons license, unless indicated otherwise in a credit line to the material. If material is not included in the article's Creative Commons license and your intended use is not permitted by statutory regulation or exceeds the permitted use, you will need to obtain permission directly from the copyright holder.
To view a copy of this license, visit https://creativecommons.org/licenses/by/4.0/

Response to Reviewers

Manuscript: NCOMMS-25-79200A-Z, "Molecular rotors reveal the 3D viscous habitat of mucus-colonizing bacteria"

Note: The reviewer comments are reproduced verbatim below, in italics. Our responses are non-italicized.

Reviewer 1

In this paper, Inman et al, have described a method to quantify and map viscosity of 'undisturbed mucus and associated bacteria' using a viscosity-sensing fluorescent ratiometric probe, RY3. Further the authors have applied their method in mucosa produced by two diatom species as well as mucosa surrounding upside down jelly fish. Further authors have attempted to link viscosity of the mucosa to specific aspects of the organisms. Authors state that "Individual mucus aggregates from cultures of Chaetoceros affinis and Pseudo-nitzschia sp. diatoms exhibit consolidated versus patchy viscous morphologies, respectively, that result in distinct patterns of microbial colonization." Authors also find that mucus surrounding upside down jellyfish Cassiopea xamachana to be viscous such that a microbiome is maintained while minimizing microbial contact. For their work, authors examine the blue and red emission from the previously described molecular rotor dye RV3 and describe a ratiometric readout. It is to be noted that the raw ratiometric readout is not suitable due to confounding factors like autofluorescence and hence they attempt to develop a calibration curve in-vitro using water-glycerol mixtures and then extending this technique to mucus in three distinct biological contexts.
While the paper presents an interesting and welcome application of a micro-environment sensitive fluorescence probe to 'undisturbed' mucosa surrounding distinct biological entities, several open questions remain. Specifically there appear to be important caveats and potential limitations of the fluorescence probing/ratiometric approach that are not addressed in the opinion of this reviewer. Therefore it is not clear that the readout being studied is faithfully reporting the viscosity of the mucus. It is plausible the readout is being convoluted by other complicating factors like polarity, differential autofluorescence and chemical composition of the media as well differential scattering from the 3-D bioaggregates. Further, in the absence of supporting experiments through independent methods and specific validation of the probe in complex, microheterogeneous media, the data presented in this paper may not support conclusions presented here. Specifically the study notes correlations of bacterial penetration and colonisations with differential viscosity readouts as described here. It is not clear whether these are incidental correlations or if there is a causal relationship between 'viscosity' and any aspects of the complex mucosa.

> **Reviewer 1 Summary**: The reviewer expresses concerns with the reliability of our method in complex, chemically-heterogeneous mucus material. Their constructive points are addressed with additional tests, calibrations, and comparison with an independent microrheological technique in the responses below. In brief, we now provide calibrations of the molecular rotors in solutions of varying complexity that are all fit by the same power law (Fig. R7 below), demonstrating that our findings do not depend on the composition or complexity of the calibration solutions. Critically, the relative viscosity probabilities we measured in *C. affinis* aggregates strongly agree with multiple particle tracking microrheology (MPTM) measurements of aggregates from the same species published in a separate study (Guadayol et al. 2021; Fig. R4 below). These separate lines of evidence better establish the reliability of our mucus viscosity measurements with molecular rotors and support our conclusions.

Specific points:
1. Differential and changing contributions from mucosa autofluorescence to the red and blue channels would significantly compromise the reporting of viscosity from the dye, using the method as described here. This is particularly relevant when comparing viscosity in two distinct biological milieu, wherein the contribution of autofluorescence may be very different.

We thank the reviewer for raising this point. The only autofluorescence with 404 and 488 nm excitation in mucus from the diatoms and upside-down jellyfish (zooxanthellae) is consistent with Chl a / PSII with a peak at 685 nm (Fig. R1A). This was verified in control samples in the absence of dyes. No blue autofluorescence was present in these samples. There is spectral overlap between red RY3 and Chl a emissions, with a 35 nm gap between the two peaks (Fig. R1B). However, this represents greater peak separation and less overlap than in other publications that utilize linear spectral unmixing (e.g. Haraguchi et al. 2002; Kato et al. 2002; Levenson & Mansfield 2006; Nadrigny et al. 2006; Schulte 2024). Indeed, only 4% of the Chl a maximum intensity is present at the RY3 peak at 650 nm (Fig. R1B). All imaging was performed with a 32 channel spectral detector (10 nm bins from 400-720nm), providing high spectral resolution for unmixing. The Chl a spectral profile was determined from control samples, and the RY3 spectral profile was determined from viscosity standards (no difference in shape between different viscosity standards or dye concentrations). Experimental images were acquired with 4x line averaging to improve the signal-to-noise ratio and spectral unmixing performance. The Chl a and red RY3 profiles were spectrally unmixed from all experimental images with a high degree of accuracy given the spectral resolution, experimentally-determined profiles, consistent optical configuration, and line averaging (Zimmermann 2005; Zimmermann et al. 2014). This procedure accounts for varying contributions of Chl a autofluorescence within and across experimental images on a pixel-by-pixel basis.

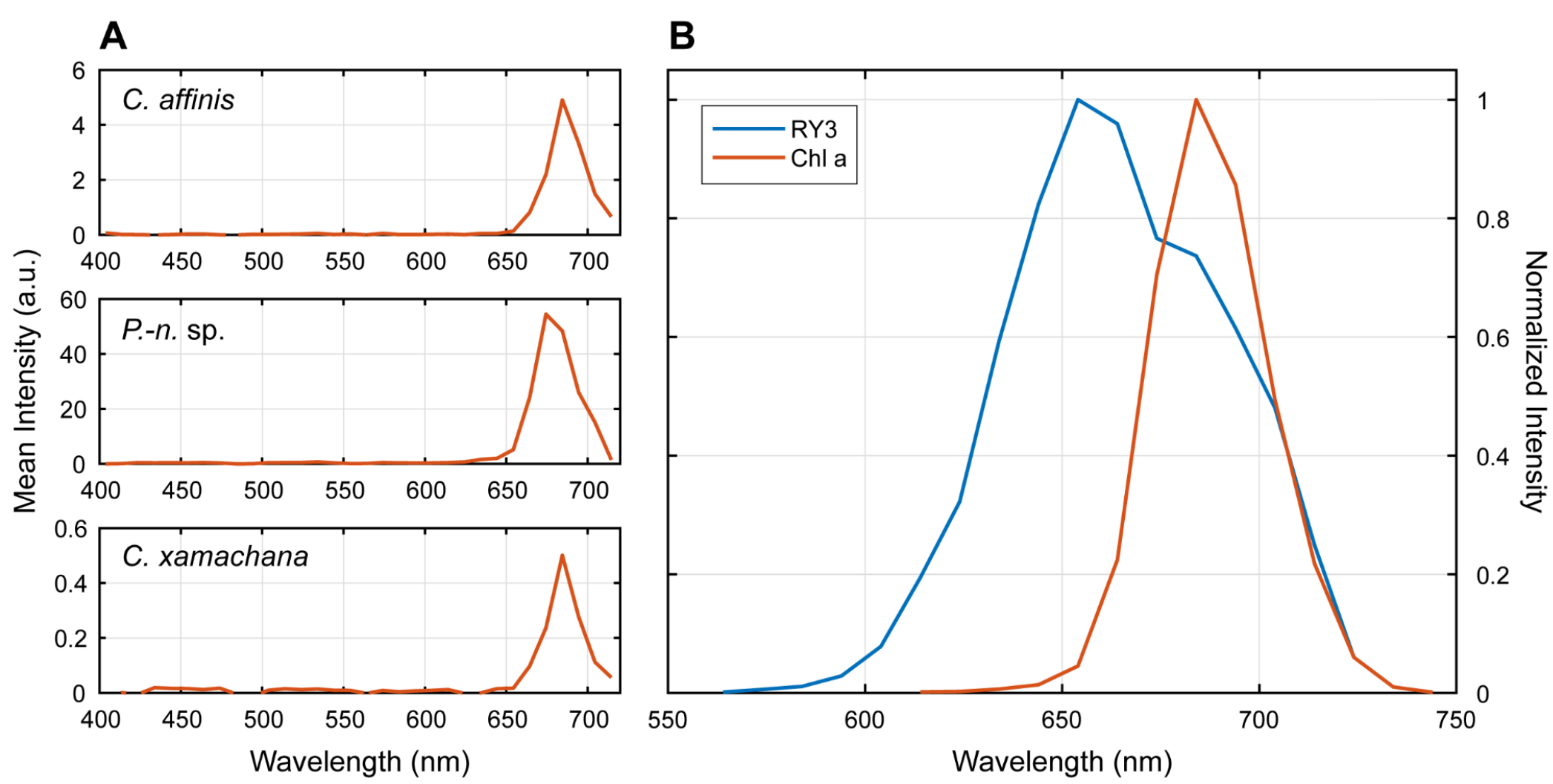

Fig. R1. (**A**) Autofluorescence spectra of mucus sampled from *C. affinis*, *P.-n.* sp., and *C. xamachana*. Excitation was 405 and 488 nm, the same as in all experiments. (**B**) The characteristic red RY3 and Chl a spectra used for unmixing experimental images.

To quantify the contribution of Chl a autofluorescence to the unmixed RY3 signal, we can spectrally unmix control images of *C. affinis* diatoms with no dyes and compare the percentage of signal that ends up in the unmixed RY3 channel versus the unmixed Chl a channel (e.g. Fig. R2). Only 0.44 ± 0.089 % SD of Chl a intensities (N=3, Fig. R2B) end up in the RY3 channel (Fig. R2C) after unmixing. In our

experimental samples, Chl a is mostly localized to the chloroplasts of diatoms or zooxanthellae (in the case of the jellyfish ephyra). Because we remove these cells in the regions we analyze, there is only a relatively small amount of Chl a present in the analyzed mucus that contributes negligible signal (0.44%) to the unmixed RY3 channel.

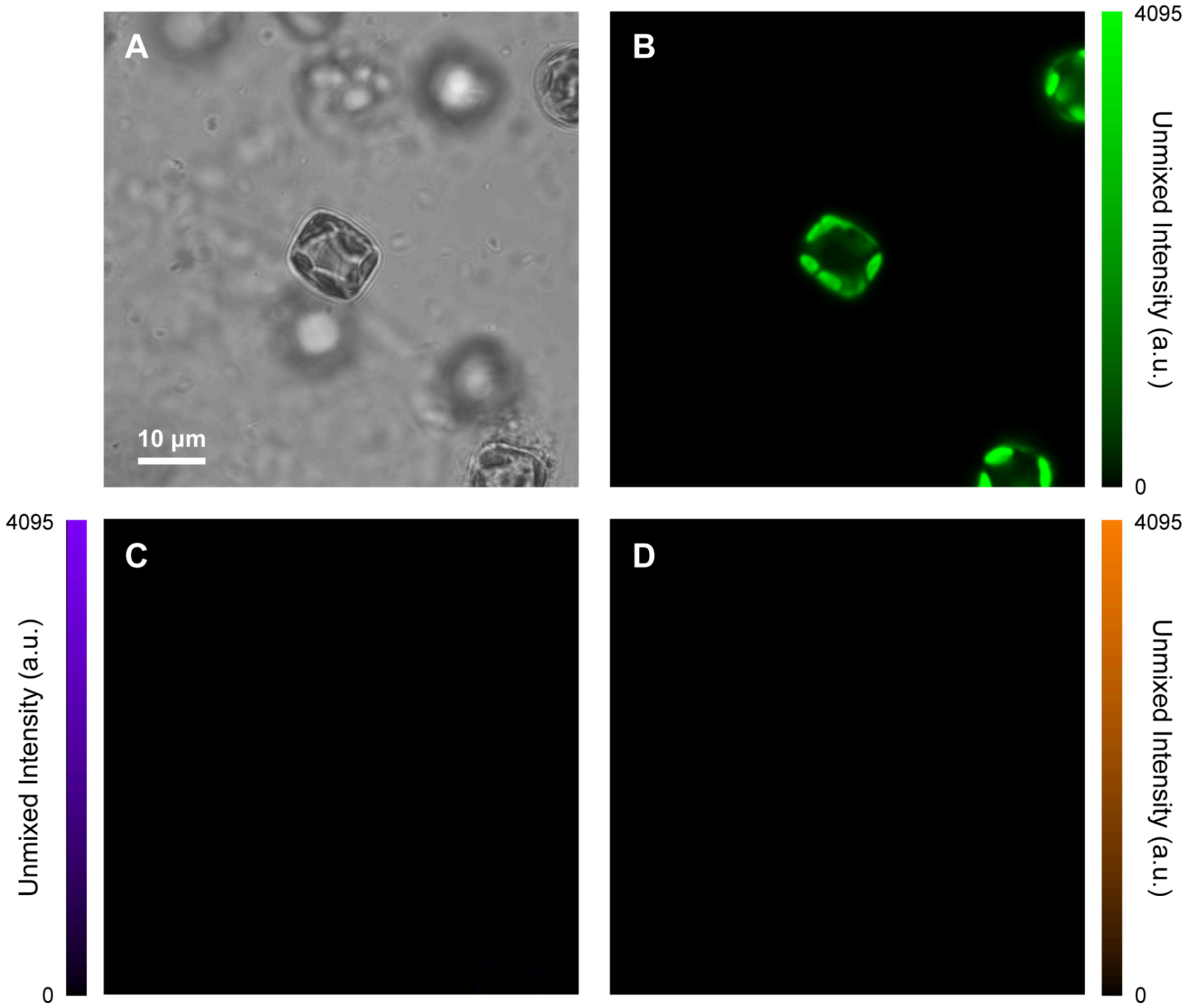

Fig. R2. A spectrally-unmixed image of *C. affinis* diatom cells with 404 and 488 nm excitation and no dyes. (**A**) Transmitted light showing the locations of the three centric diatom cells. (**B**) The unmixed Chl a channel in green. (**C**) The unmixed RY3 channel in purple. (**D**) The unmixed SYBR Green I channel in orange. Only green is visible, localized to the chloroplasts of the diatom cells. Trace Chl a is present in mucus material located above the diatom on the bottom right.

Revisions: Added lines 418-419 in the Main Manuscript. Added lines 636-670 and Fig. S4-S5 in the Supplementary Information.

2. An important finding of the paper as described here is the comparison of viscosity from two different mucosa surrounding distinct diatom species. Clearly the chemical composition would be different. It is not clear from this study if confounding factors like specific chemical interactions, polarity and factors other than viscosity would differentially effect the blue and red channels. This needs to be addressed in detail.

We thank the reviewer for making this important point. While is it not possible to test specific chemical interactions between RY3 and the 10^4-10^6 estimated organic compounds present in phytoplankton exudates (Catalá et al. 2021), calibrations of RY3 with different classes of molecules and comparison with an independent microrheological technique verify the reliability of our ratiometric method in complex

biofluids. These separate lines of evidence demonstrate that the differential influence of chemical interactions on the blue and red channels of RY3 is negligible.

Specific chemical interactions: In addition to glycerol, we had performed calibrations of RY3 with Ficoll (400K mw) and dextran (5-40M mw) solutions, both complex fluids with highly-branched polysaccharides. The normalized fluorescence intensity ratios (glycerol, Ficoll) and fluorescence lifetimes (Dextran) as a function of macroviscosity are fit by a single power law (Fig. R7), proving that our results are not influenced by the complexity of the calibration solutions (see Point 7 below for details). Peng et al. (2011) tested RY3 in solutions of the protein BSA and the amino acid cysteine, and found that changes in "fluorescence intensity, and fluorescence ratio are all negligible". They also assayed RY3 in solutions with dielectric constants that ranged from 7.43 to 78.36 and concluded that "the polarity of the solvents has little effect on fluorescence quantum yields." The ratio of red to blue quantum yields was 6.6 ± 1.9 SD (N=6) and did not significantly vary with dielectric constant, confirming that the differential influence of polarity on the RY3 red and blue channels is negligible. It should also be noted that the diatom aggregates and jellyfish mucus are >95% seawater by mass (Passow 2002; Hubot et al. 2022), and so polarity is not expected to vary substantially in these samples.

Comparison with an independent microrheological technique: It is not trivial to intercalibrate molecular rotors and other microrheological techniques (e.g. MPTM) in mucus aggregates: microspheres do not readily penetrate mucus and their Brownian motion interferes with time-averaged RY3 fluorescence imaging. However, we can compare viscosity measurements of mucus aggregates from the same diatom culture (*Chaetoceros affinis*) using MPTM (Guadayol et al. 2021, Fig. R3 C and D) and molecular rotors (our study, Fig. R3 A and B).

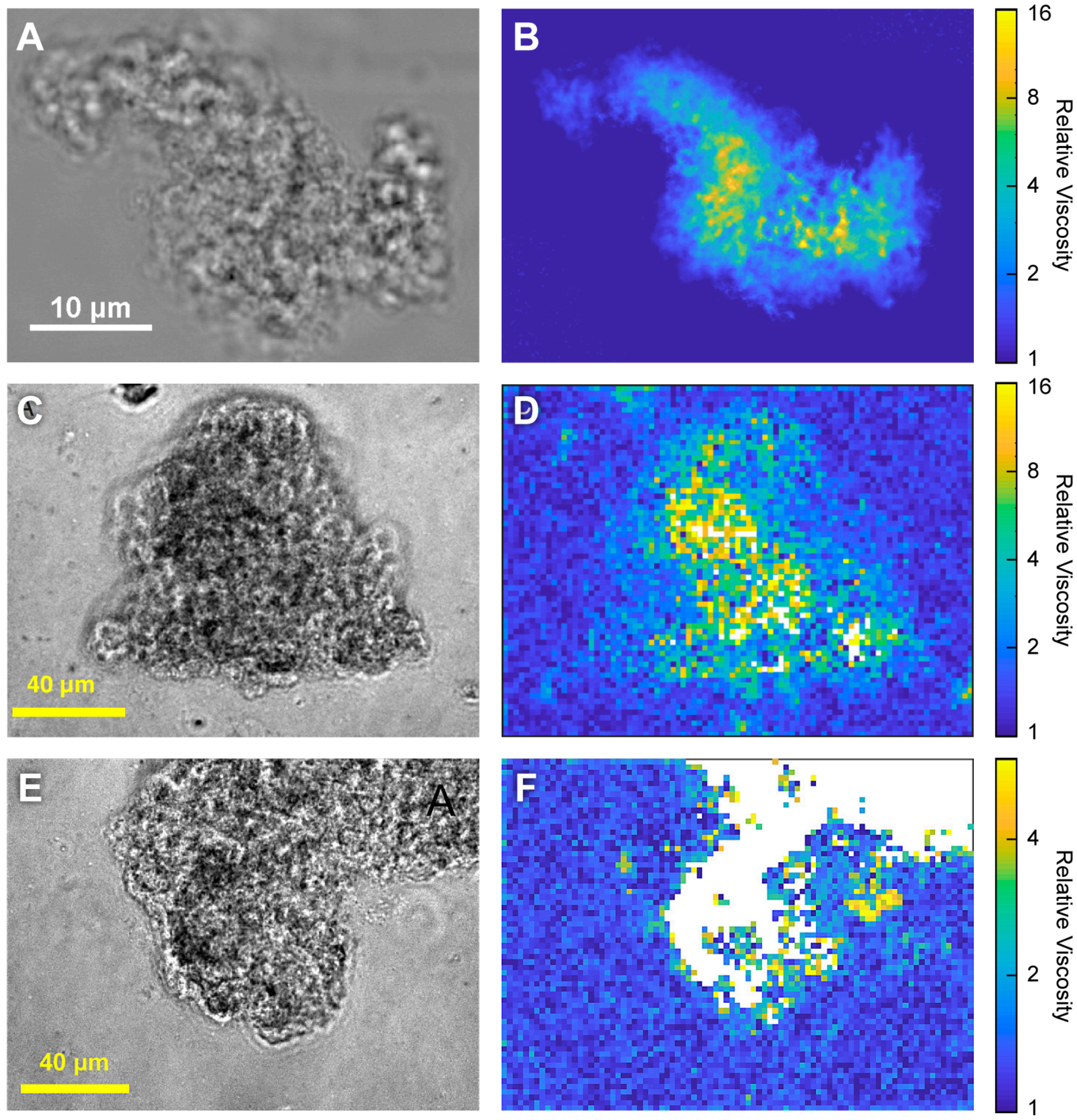

Fig. R3. Comparison of *C. affinis* aggregates measured with molecular rotors and MPTM. Transmitted light (**A**) and relative viscosity via RY3 (**B**) from this study. Phase contrast (**C**) and relative viscosity via MPTM (**D**) in a focal plane close to the edge of the aggregate in the z-direction from Guadayol et al. (2021). White pixels are no data, indicating microspheres stuck in the mucus. (**E** and **F**) The same as (C and D) but for a different aggregate showing typical microsphere penetration ~1/3 of the way into the aggregate in Guadayol et al. (2021). Both studies used the same culturing media and similar culturing conditions.

There is strong agreement between the ranges of relative viscosities in aggregates estimated from the two techniques (N=8 in Guadayol et al. 2021; N=30 this study). The pattern of viscosity increasing toward the center of the aggregate is also consistent. Further, **the values and probabilities of relative viscosities in aggregates measured with MPTM corroborate our ratiometric molecular rotor measurements** (Fig. R4).

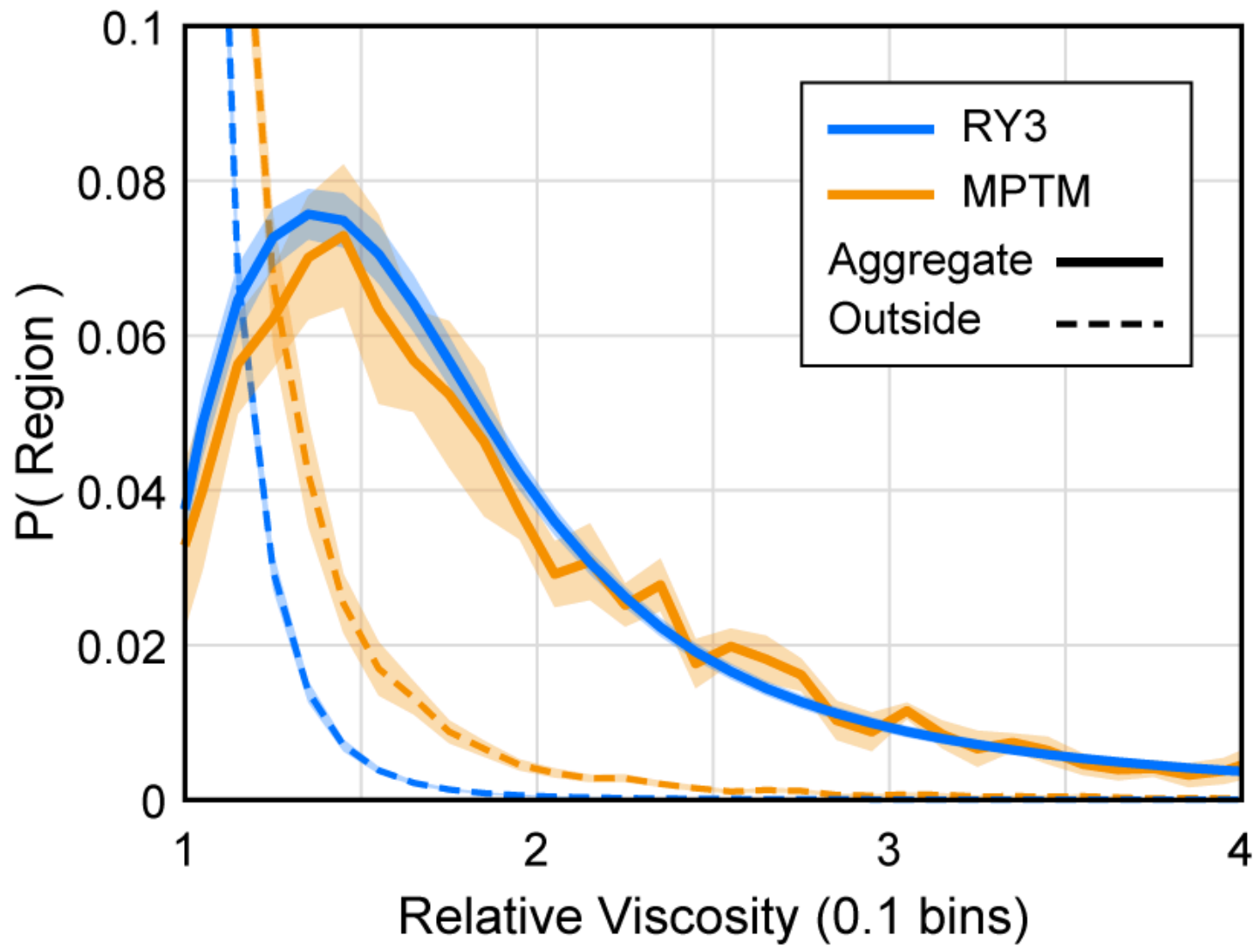

Fig. R4. The probabilities of relative viscosities from the edge regions (solid lines) or outside (dashed lines, control) of *C. affinis* aggregates, measured with MPTM (orange, Guadayol et al. 2021, N=8 aggregates) and RY3 molecular rotors (blue, this study, N=30 aggregates). The most probable relative viscosity in both MPTM and RY3 outside regions is 1 (seawater). Shaded areas represent the standard error.

The MPTM measurements in Guadayol et al. (2021) only represent the outer ~1/3 edges of the aggregates (Fig. R3F), as the authors state "the spatial coverage of the MPTM maps inside the aggregates is limited by the capacity of the microspheres to penetrate the aggregates." For comparison with our molecular rotor measurements, we can select data from edge regions which are defined as pixels from the outer boundary inward 1/3 of the maximum distance inside the aggregate. This definition accommodates complicated aggregate geometry and matches microsphere penetration in Guadayol et al. (2021). The relative viscosity probability distributions of our edge regions show excellent agreement with the MPTM measurements in Guadayol et al. 2021 (Fig. R4). A two-sided permutation test of Wasserstein distances to determine if the MPTM and RY3 data come from different distributions results in p=0.49 and T_{obs}=-0.051, indicating that the MPTM and RY3 distributions are statistically indistinguishable. If "specific chemical interactions, polarity and factors other than viscosity would differentially effect the blue and red channels", the RY3 viscosity probabilities would not match MPTM, an independent technique that relies on fundamentally different physical principles. Instead, **this agreement between RY3 and MPTM aggregate viscosity probabilities shows that our viscosity measurements are not significantly influenced by the composition, heterogeneity, or complexity of real mucus material.**

Revisions: Added lines 114-121 in the Main Manuscript. Added lines 573-635 and Fig. S1-S3 in the Supplementary Information.

3. Authors compare FLIM and ratiometric approaches. Authors have claimed that "it is difficult or impossible to separate overlapping autofluorescence with FLIM". They have referred to the Supplementary information section to justify their claim but surprisingly no quantifiable arguments have been provided. What was the contribution from AF of Chlorophyll and related pigments in the red emission region of RY3? This is an important factor that is not fully addressed. In general AF have lower lifetime and needs high laser power to image and therefore can easily be separated from dye lifetime either by using low laser power or by careful analysis using gating.

Chlorophyll can have a range of lifetime depending on whether its in free or bound form. It would have been instructive to see the AF distribution in the used experimental setting.

We appreciate the reviewer's viewpoint. Prior to conducting our study, a review of the literature on autofluorescence in diatoms indicated that lifetimes could range from 0.13 to 5.7 ns (Miloslavina et al. 2009; Zeng et al. 2012; Cvjetinovic et al. 2020). Our lifetime measurements of RY3 in dextran solutions ranged from 0.15 to 0.35 ns (Fig. R7C). Due to the predicted overlap of RY3 and diatom autofluorescence lifetimes– as well as limited access to a FLIM system – we did not measure autofluorescence lifetimes in our study species. It is also important to note that FLIM systems are more expensive and rare compared to conventional confocal microscopes, which is why it is important to provide ratiometric methods for the broader community. Therefore, this manuscript focuses on ratiometric intensity measurements instead of FLIM. We have changed our discussion of FLIM accordingly.

Autofluorescence intensity measurements are provided in Fig. R1.

Revisions: Added line 305 in the Main Manuscript. Removed text in the "FLIM vs. ratiometry" section (line 678) of the Supplementary Information.

4. The authors use exclusively use RY3 for the purpose of quantifying viscosity. Rationale is not clear. For instance There are ratiometric and FLIM dyes (for instance see Yang et al, JACS 2013) for viscosity with emissions at blue and green region that could have been used to avoid AF at far red region if there was a huge contribution from AF. In that context some complex analysis and calibration steps mentioned in the paper could have been avoided.

We demonstrate in the response to Point 1 and Fig. R1 that the contribution of autofluorescence to the RY3 red channel is minimal in our system. The ratiometric and FLIM dye "compound 1" (C1) in Yang et al. (2013) has emission maxima at 427 and 516 nm. Unfortunately, the green peak of C1 has complete overlap with SYBR Green I (520 nm), which was required in our study for locating bacterial cells. Such an overlap cannot be easily separated with spectral unmixing. C1 is also less sensitive than RY3 in the viscosity range 1-10 mPa·s relevant to diatom and jellyfish mucus. However, we are interested in using C1 in other applications and thank the reviewer for their suggestion.

Revisions: Added lines 714-715 in the Supplementary Information.

5. RY3 is a lipophilic cationic dye and also cell permeable. No experiment has been conducted to check if RY3 could stain the colonizing bacteria. It is possible that signal that is presumed to be from mucosa alone is indeed from the bacterial membranes as well. A simple control experiment using any bacteria could have been performed to address this.

We thank the reviewer for raising this issue. If RY3 were lipophilic it would localize to the bacterial membranes, appearing as a ring of uncalibrated fluorescence surrounding (and not overlapping) SYBR Green I nucleic acid fluorescence inside the cell. Instead, confocal images of *Pseudoalteromonas* sp. TW7 bacteria show complete spatial overlap of the unmixed RY3 and SYBR fluorescences (Fig. R5). Every experimental image in the manuscript included bacteria that were not associated with mucus (i.e. 'outside' bacteria in main manuscript Fig. 3A and 4C), and they exhibited the same spatial overlap of RY3 and

SYBR. This demonstrates that RY3 does not preferentially localize to cell membranes, and therefore is not particularly lipophilic.

Furthermore, we included a relatively large spatial buffer in our bacterial detection algorithm to prevent any RY3 signal in the bacterial cells from entering our analysis of mucus. The bacteria in our experiments were generally ~1 µm diameter, and the SYBR signal extended most of the way to the cell membrane. We dilated the SYBR signal by 0.4 µm in all directions and removed the resulting volumes from our mucus analyses to ensure no signal overlap. This spatial buffer is the cause of the "black area between the orange circle and SYBR 1 signal (DNA) in Fig. 2 and Fig. 4" that the reviewer notes in Point 10 (below).

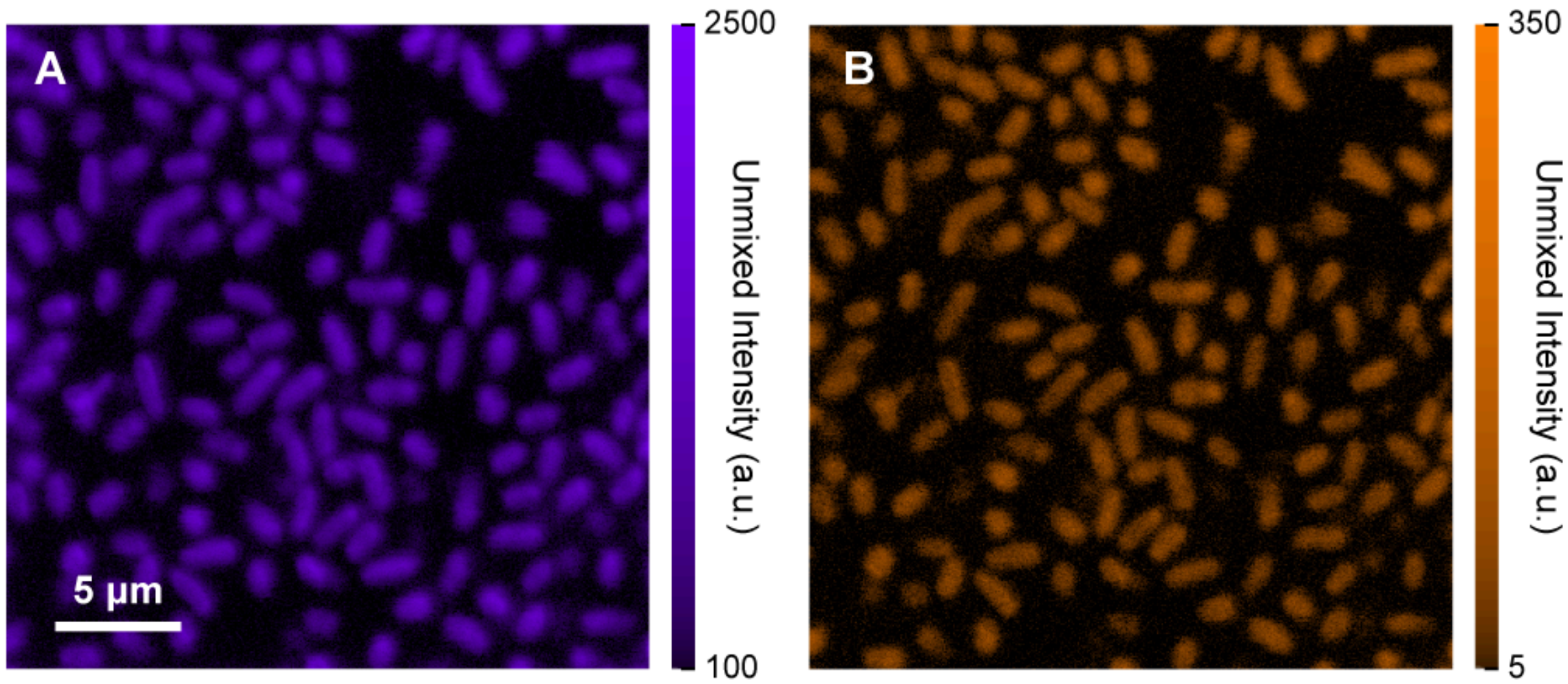

Fig. R5. A confocal microscopy thin section of unmixed RY3 (A) and SYBR Green I (B) fluorescences from a culture of *Pseudoalteromonas* sp. TW7 bacteria.

Revisions: Added lines 742-752 and Fig. S6 in the Supplementary Information.

6. The concentration of RY3 used for this study is very high compared to the original paper. Such amphipathic dyes are known to self-aggregate and form nanocluster where its photophysics might change. It would have been nice to provide fluorescence spectra for the dye concentrations mentioned at Fig. 1A. Additionally, a control experiment of imaging the dye in solution can also give information about self-aggregation induced formation of nano-aggregates.

We thank the reviewer for raising this point. RY3 fluorescence spectra for the blue and red peaks do not exhibit peak shifts, new peaks, or reduced quantum yields with a 16-fold increase in dye concentration (Fig. R6 A and B). This demonstrates that the photophysics of the rotor are not influenced by high concentrations and that nano-aggregates are not likely present. No aggregates are visible in confocal microscopy images of 800 µM RY3 (e.g. Fig. R6C), although it is not clear that we could resolve nano-aggregates with our microscope. Regardless, high concentrations of the dye do not influence our results, and ratiometric calibration provides quantitative viscosity measurements that are independent of dye concentration.

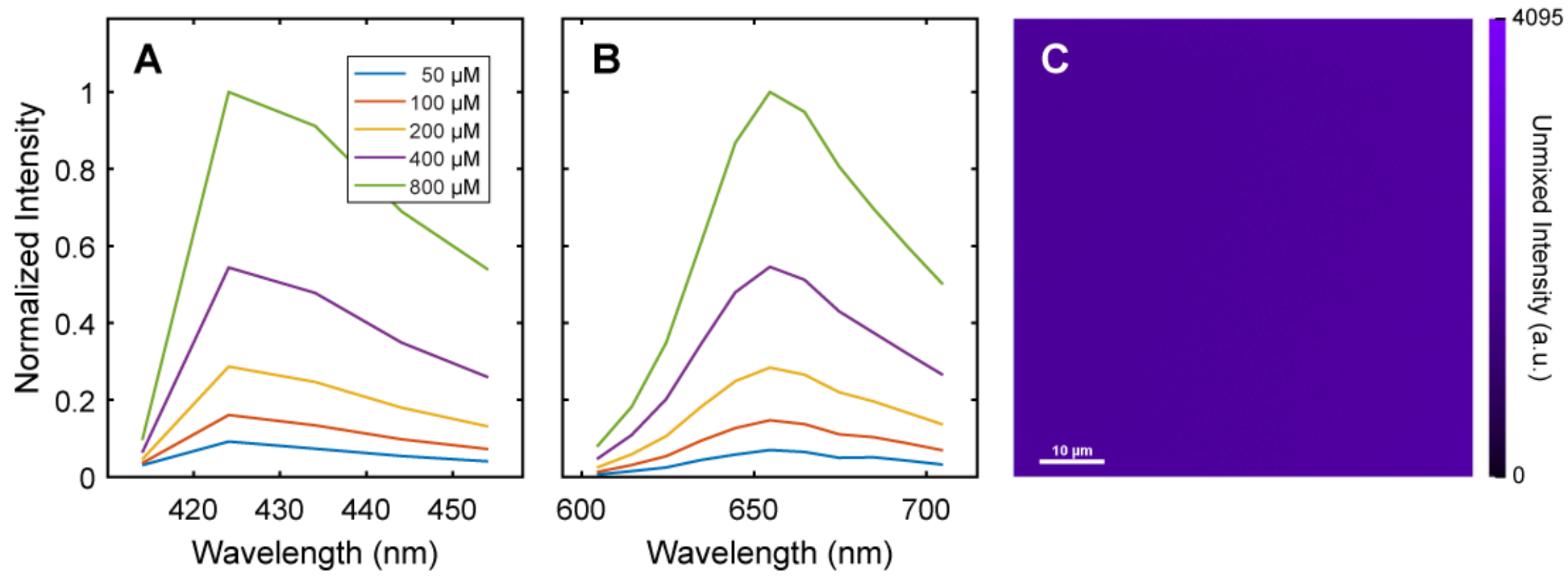

Fig. R6. Fluorescence spectra of varying concentrations of RY3 in 18.25 relative viscosity glycerol solutions. The spectra are normalized by the blue (**A**) or red (**B**) maxima for visualization. (**C**) A confocal microcopy image of unmixed RY3 fluorescence in 800 µM RY3.

Revisions: Added lines 364-365 in the Main Manuscript. Added lines 791-801 and Fig. S8 in the Supplementary Information.

7. The whole technique rests on a calibration curve that is developed in solution. This complex calibration curve is then used in highly micro-heterogeneous mucus and a comparison is made between putative viscosity readouts from distinct mucus samples originating from distinct bio-specimens/species. Directly relating calibration curves obtained in solution/solvents to highly complex and varied media is fraught with risk. Validation of the calibration curves in controlled samples (amenable to systematic modulation of viscosity) and that resemble the mucus would need to be performed.

We agree that this is an important consideration. We are not aware of controlled biological samples similar to diatoms and jellyfish in which viscosity could be systematically modulated, as the reviewer suggests. Instead, validation of our molecular rotor method in complex biofluids is best demonstrated by agreement with an independent microrheological technique (see Point 2 and Fig. R4, above). We also provide additional calibrations of RY3 in polymer solutions to show that our results are not influenced by the rheological complexity of the solution (Fig. R7).

We imaged the fluorescence intensity of RY3 in aqueous solutions of glycerol (rheologically simple) and Ficoll 400 (semi-complex, 400K mw) on a confocal microscope. The ratio of red to blue fluorescence of RY3 facilitates comparison of the two viscous agents in a consistent manner. The macroviscosity of these solutions were measured on a rheometer with a concentric cylinder geometry. We performed Fluorescence Lifetime Imaging Microscopy (FLIM) of aqueous solutions of dextran (complex, 5-40M mw) on a different confocal microscope and measured the macroviscosity of those solutions on a different rheometer with a cone and plate geometry. Normalizing the three calibrations by their values at the highest common macroviscosity, they all fall onto a single curve that is fit by a power law (Fig. R7). **This demonstrates that the fundamental relationship between our RY3 measurements and macroviscosity does not depend on the rheological complexity of the calibration solution.**

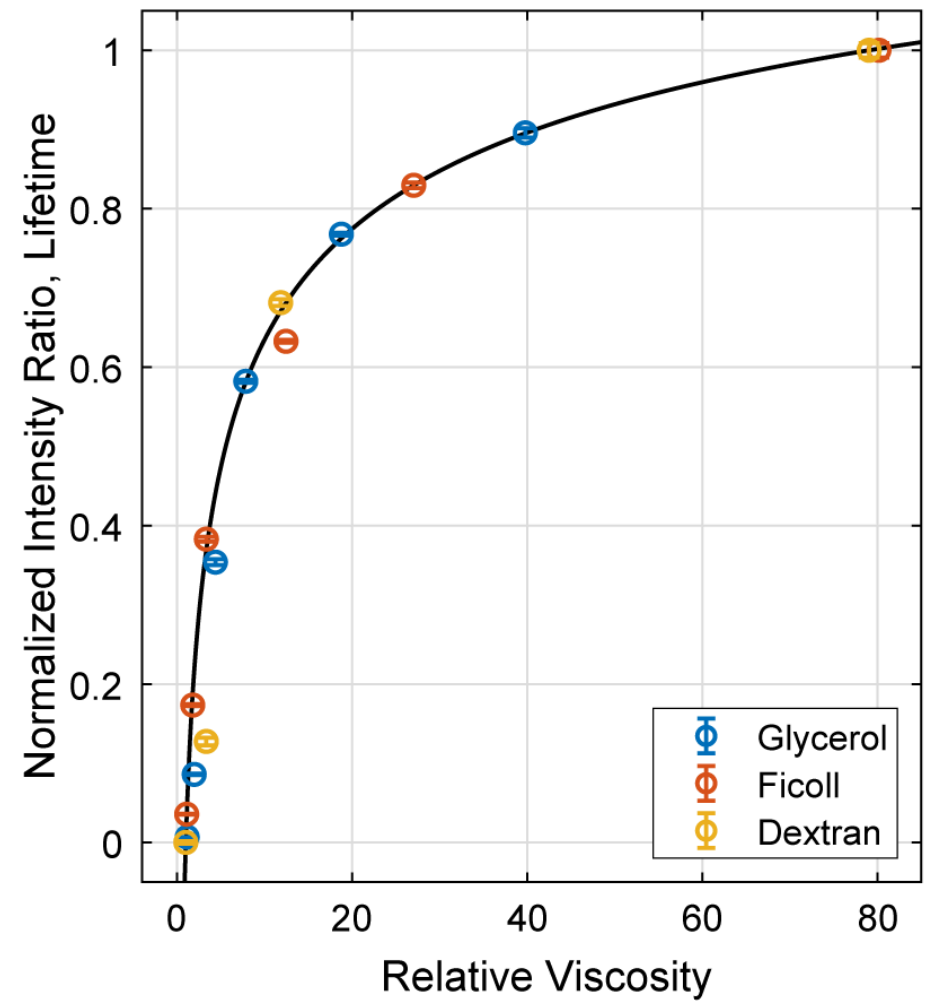

Fig. R7. The RY3 red/blue fluorescence intensity ratio (glycerol, Ficoll) and fluorescence lifetime (dextran) as a function of relative macroviscosity, normalized by their values at the highest common relative viscosity (interpolated between relative viscosity 40 and 275 for glycerol). All three are fit by the power law $x^{-0.2}$ with R^2=0.992 and RMSE=0.037 (black line).

Revisions: Added lines 114-121 and 321-323 in the Main Manuscript. Added lines 618-635 and Fig. S3 in the Supplementary Information.

8. A significant aspect of this study is the comparison of the viscosities from the two diatom mucosa and how this may explain differential bacterial penetration and colonizing. Are there other explanations of differential penetration and colonizing. What is the evidence that viscosity is the causal factor? Can such correlations arise due to other factors?

We thank the reviewer for bringing up these questions. There are many factors that could influence bacterial colonization of mucus, such as binding of surface-expressed lectins, bacterial motility, or elastic properties of the mucus. Our study only demonstrates a correlation between bacterial location and mucus viscosity, not a causal relationship. We have changed the language in our manuscript to better reflect this point. It should be noted that our study is the first to map a physical parameter in 3D throughout the interior of undisturbed mucosa, allowing quantification of bacterial colonization of a physical structure. Combining this method with other assays will enable future research to determine a causal relationship between bacterial colonization and mucus properties, and our study provides a critical starting point.

Revisions: Modified lines 159, 168, 196, 212 and added lines 276-282 in the Main Manuscript.

9. It is not clear how scattering from mucosa may differentially affect the blue and red channels and how this may change depending on the context. This may significantly compromise the calibration used here.

If scattering from mucosa differentially affected the blue and red channels, both the spatial patterns (Fig. R3) and viscosity probability distributions (Fig. R4) of aggregates would substantially differ between our method and an independent microrheological technique (see Point 2, above). Instead, measurements of natural mucosa with the two techniques agree, confirming that our results are not significantly influenced by differential scattering. Further, we experimentally determined the point spread function (PSF) of our microscope and deconvolved it with all channels in experimental data to reduce scattering effects.

Revisions: Added lines 573-617 and Fig. S1-S2 in the Supplementary Information.

10. Minor point: Further, this reviewer can see black area between the orange circle and SYBR 1 signal (DNA) in Fig. 2 and Fig. 4. No explanation has been offered for this.

We included a spatial buffer in our bacterial detection algorithm to prevent any RY3 signal in the bacterial cells from entering our analysis of mucus (see Point 5, above). This spatial buffer appears as a small black gap between the SYBR signal (green) and mucus near the bacteria (orange) in Fig. 2 and 4.

Revisions: Added lines 742-752 and Fig. S6 in the Supplementary Information.

Overall while this is an interesting attempt, there appears to be significant caveats that are not fully addressed here. Also this is a study that presents potentially interesting correlations but it's not clear how it may be possible to definitively link viscosity as the key factor that governs specific features of mucosa especially when comparing diverse samples.

Reviewer 2

I co-reviewed this manuscript with one of the reviewers who provided the listed reports. This is part of the Nature Communications initiative to facilitate training in peer review and to provide appropriate recognition for Early Career Researchers who co-review manuscripts.

Reviewer 3

This is an intriguing manuscript that describes the locations of bacteria and phytoplankton in three contexts. The use of the molecular rotor RY3 seems novel and some insights about the distribution of these microorganisms has been provided. I have some concerns that need addressing to be more certain about the manuscript's conclusions.

Major comments:

How many times was the calibration presented in figure 1 repeated? If only once what is the justification.

We thank the reviewer for raising this question. The glycerol-based calibration in Fig. 1 was repeated four times with a similar configuration on the same microscope, with minor modifications. Modifications between calibrations included varying the distance from the bottom of the slide at which images were captured, increasing the number of dye concentrations, and testing new RY3 dye stocks. The shape of the calibration curves did not change between calibrations. Additionally, we performed calibrations with Ficoll 400 (400K mw) with the same microscope and Dextran (5-40M mw) with a different FLIM microscope (see Fig. R7 and associated discussion). The shapes of all calibration curves were the same, demonstrating that our calibration does not depend on the complexity of the viscosity standard solutions or the microscopy setup. Within each calibration, the reported value is the mean of three images (each representing 1,048,576 point measurements of fluorescence intensity) taken at different horizontal locations. The error bars are smaller than the markers for each data point and are visible in Fig. R7. Agreement between measurements of diatom aggregates using RY3 and an independent microrheological

technique confirms the reliability of our method in complex biofluids (see Fig. R4 and surrounding discussion).

A full calibration of the method from making viscosity standards through image analysis is an extensive and comprehensive process performed over ~1 month. After calibration, a pair of viscosity standards are imaged alongside every experimental microscopy session. These standard images are used in the calibration of experimental images to account for any optical or environmental variability between sessions, since a full calibration is not possible for each experimental imaging session.

Revisions: Added lines 114-121 in the Main Manuscript. Added lines 573-635 and Fig. S1-S3 in the Supplementary Information.

Why was only 1 aggregation analyzed for each diatom? I understand that many microorganisms were looked at within the aggregation, but I am perplexed that 1 aggregation is enough to rule out chance playing an outsized role here.

We acknowledge and appreciate the reviewer's point. We attempted to publish a previous version of this manuscript that both introduced the method and examined population-level differences in mucus aggregates. This was rejected because the reviewers did "not think that the authors have sufficiently established the method of molecular rotors for this application," which would go over the word-count limit of the journal. Therefore, the goal of this manuscript is to introduce the molecular rotor method and provide "proof of concept" examples of how it can be used to extract quantitative viscosity data from individual mucus samples, rather than quantify consistent differences between aggregates from two populations of diatoms. This is the first work that maps a 3D physical parameter and bacterial associations in undisturbed mucus material, with more than 10^6 point measurements of viscosity in each sample. As such, we chose to showcase the method and analyses using individual aggregates rather than making claims about statistically significant differences between treatments (which would require more replicates). There is a precedent for this type of 'concept paper' in Nature Communications, which focuses on a method and uses individual examples (e.g. Lyons et al. 2023, Fig. 2-4; Dong et al. 2025, Fig. 3-5; Bílá et al. 2026, Fig. 2-3). Our method took 4 years to develop and calibration / analysis of each 3D aggregate is expensive in time and computational resources. As such, the examples presented in this manuscript represent the culmination of comprehensive efforts to adequately showcase the utility of the method without making claims about statistically significant differences between treatments. Rather, this will be the focus of subsequent work examining replicate aggregates from different populations, once the method has been published.

Revisions: For clarity, modified lines 36, 146-148 in the Main Manuscript.

It seems that only 1 Cassiopea ephyra was analyzed in figure 4. Is this correct? If so, why not improve the rigor with some reproduction of the results?

See the response to the previous comment. The purpose of the manuscript is to showcase the novel method and analyses with individual examples derived from different biological sources, not to demonstrate consistent effects between treatments for which replicates would be required.

Revisions: For clarity, modified line 223 in the Main Manuscript.

Minor comments:

It is worthwhile to describe marine snow directly in the introduction. It may be useful for a more general audience.

Thank you for the suggestion, we have expanded our description of marine snow in the introduction.

Revisions: Added lines 46-49 in the Main Manuscript.

Should the title be more inclusive of diatoms?

Since the manuscript examines an animal (jellyfish) as well as unicellular algae, we feel that including diatoms in the title would be too narrow. Similarly, including both diatoms and jellyfish to the title would make it too long. Our main focus is how molecular rotors reveal the 3D viscous habitats of bacteria, so we feel keeping the title general is most effective and appropriate for the Nature Communications audience.

References

Bílá, Š., Pinkas, D., Khakurel, K., Boger, J., Bílý, T., Hajdu, J., Franta, Z., de Diego Martinez, I., Tuma, R., Redecke, L., & Polovinkin, V. (2026). Single-cell structural biology with intracellular electron crystallography. Nature Communications, 17(1), 2109. https://doi.org/10.1038/s41467-026-69205-6

Catalá, T. S., Shorte, S., & Dittmar, T. (2021). Marine dissolved organic matter: A vast and unexplored molecular space. Applied Microbiology and Biotechnology, 105(19), 7225–7239. https://doi.org/10.1007/s00253-021-11489-3

Cvjetinovic, J., Salimon, A. I., Novoselova, M. V., Sapozhnikov, P. V., Shirshin, E. A., Yashchenok, A. M., Kalinina, O. Yu., Korsunsky, A. M., & Gorin, D. A. (2020). Photoacoustic and fluorescence lifetime imaging of diatoms. Photoacoustics, 18, 100171. https://doi.org/10.1016/j.pacs.2020.100171

Dong, Z., Zhou, H., Cao, R., Zhang, O., Zhao, S., Lyu, P., Alcalde, R., & Yang, C. (2025). Analytic Fourier ptychotomography for aberration-free and high-resolution volumetric refractive index imaging. Nature Communications, 17(1), 727. https://doi.org/10.1038/s41467-025-67460-7

Guadayol, Ò., Mendonca, T., Segura-Noguera, M., Wright, A. J., Tassieri, M., & Humphries, S. (2021). Microrheology reveals microscale viscosity gradients in planktonic systems. Proceedings of the National Academy of Sciences, 118(1), e2011389118. https://doi.org/10.1073/pnas.2011389118

Haraguchi, T., Shimi, T., Koujin, T., Hashiguchi, N., & Hiraoka, Y. (2002). Spectral imaging fluorescence microscopy. Genes to Cells, 7(9), 881–887. https://doi.org/10.1046/j.1365-2443.2002.00575.x

Hubot, N., Giering, S. L. C., & Lucas, C. H. (2022). Similarities between the biochemical composition of jellyfish body and mucus. Journal of Plankton Research, 44(2), 337–344. https://doi.org/10.1093/plankt/fbab091

Kato, N., Pontier, D., & Lam, E. (2002). Spectral Profiling for the Simultaneous Observation of Four Distinct Fluorescent Proteins and Detection of Protein-Protein Interaction via Fluorescence Resonance Energy Transfer in Tobacco Leaf Nuclei. Plant Physiology, 129(3), 931–942. https://doi.org/10.1104/pp.005496

Levenson, R. M., & Mansfield, J. R. (2006). Multispectral imaging in biology and medicine: Slices of life. Cytometry Part A, 69A(8), 748–758. https://doi.org/10.1002/cyto.a.20319

Lyons, A., Zickus, V., Álvarez-Mendoza, R., Triggiani, D., Tamma, V., Westerberg, N., Tassieri, M., & Faccio, D. (2023). Fluorescence lifetime Hong-Ou-Mandel sensing. Nature Communications, 14(1), 8005. https://doi.org/10.1038/s41467-023-43868-x

Miloslavina, Y., Grouneva, I., Lambrev, P. H., Lepetit, B., Goss, R., Wilhelm, C., & Holzwarth, A. R. (2009). Ultrafast fluorescence study on the location and mechanism of non-photochemical quenching in diatoms. Biochimica et Biophysica Acta (BBA) - Bioenergetics, 1787(10), 1189–1197. https://doi.org/10.1016/j.bbabio.2009.05.012

Nadrigny, F., Rivals, I., Hirrlinger, P. G., Koulakoff, A., Personnaz, L., Vernet, M., Allioux, M., Chaumeil, M., Ropert, N., Giaume, C., Kirchhoff, F., & Oheim, M. (2006). Detecting fluorescent protein expression and co-localisation on single secretory vesicles with linear spectral unmixing. European Biophysics Journal, 35(6), 533–547. https://doi.org/10.1007/s00249-005-0040-8

Passow, U. (2002). Transparent exopolymer particles (TEP) in aquatic environments. Progress in Oceanography, 55(3), 287–333. https://doi.org/10.1016/S0079-6611(02)00138-6

Peng, X., Yang, Z., Wang, J., Fan, J., He, Y., Song, F., Wang, B., Sun, S., Qu, J., Qi, J., & Yan, M. (2011). Fluorescence Ratiometry and Fluorescence Lifetime Imaging: Using a Single Molecular Sensor for Dual Mode Imaging of Cellular Viscosity. Journal of the American Chemical Society, 133(17), 6626–6635. https://doi.org/10.1021/ja1104014

Schulte, S. J., Fornace, M. E., Hall, J. K., Shin, G. J., & Pierce, N. A. (2024). HCR spectral imaging: 10-plex, quantitative, high-resolution RNA and protein imaging in highly autofluorescent samples. Development, 151(4), dev202307. https://doi.org/10.1242/dev.202307

Yang, Z., He, Y., Lee, J.-H., Park, N., Suh, M., Chae, W.-S., Cao, J., Peng, X., Jung, H., Kang, C., & Kim, J. S. (2013). A Self-Calibrating Bipartite Viscosity Sensor for Mitochondria. Journal of the American Chemical Society, 135(24), 9181–9185. https://doi.org/10.1021/ja403851p

Zeng, Y., Wu, Y., Li, D., Zheng, W., Wang, W.-X., & Qu, J. Y. (2012). Two-photon excitation chlorophyll fluorescence lifetime imaging: A rapid and noninvasive method for in vivo assessment of cadmium toxicity in a marine diatom Thalassiosira weissflogii. Planta, 236(5), 1653–1663. https://doi.org/10.1007/s00425-012-1703-1

Zimmermann, T. (2005). Spectral Imaging and Linear Unmixing in Light Microscopy. In J. Rietdorf (Ed.), Microscopy Techniques: -/- (pp. 245–265). Springer. https://doi.org/10.1007/b102216

Zimmermann, T., Marrison, J., Hogg, K., & O'Toole, P. (2014). Clearing Up the Signal: Spectral Imaging and Linear Unmixing in Fluorescence Microscopy. In S. W. Paddock (Ed.), Confocal Microscopy: Methods and Protocols (pp. 129–148). Springer. https://doi.org/10.1007/978-1-60761-847-8_5

Response to Reviewers 2

Manuscript: NCOMMS-25-79200A-Z, "Molecular rotors reveal the 3D viscous habitat of mucus-colonizing bacteria"

Note: The reviewer comments are reproduced verbatim below, in italics. Our responses are non-italicized.

Reviewer 1

The authors have addressed many of the questions and concerns raised earlier.
We feel the following points may be addressed through textual changes:

1) The authors note that the observations linking microviscoslity changes to bacterial colonisation are correlative and no casual relationship can be inferred. They have appropriately added a section in the discussion.
However in the abstract they state :
" Individual examples of mucus aggregates from cultures of Chaetoceros affinis and Pseudo-nitzschia sp. diatoms exhibit consolidated versus patchy viscous morphologies, respectively, that result in distinct patterns of microbial colonization."
This implies causality, in fact explicitly stating that 'distinct patterns ..of microbial colonisation' result from patchy viscous morphologies. This needs to be toned down, wherein the finding of patchy morphologies needs to be described ALONG WITH specific patterns of microbial colonisation

> We agree with the reviewer and have edited the text to say "…exhibit consolidated versus patchy viscous morphologies, respectively, **along with** distinct patterns of microbial colonization" as the reviewer requested (line 38).

2) The authors have presented detailed arguments on methodology in the response. These reviewers feel it would be beneficial to include a short section in the discussion where the caveats and assumptions underlying. the methodology used may be articulated. This section may also mention other potential ways to address viscosity (like FLIM) and these methods may be briefly compared. FLIM for instance, is independent of intensity fluctuations (even potentially autofluorescence contributions through lifetime gating) but requires specialised equipment and so on.

> We have added the following text to the Discussion section on lines 236-238 to highlight caveats and assumptions underlying the method: "The method presented here measures microviscosity at the scale of the molecular rotor; this is not the same as hydrodynamic viscosity measured with a rheometer and does not include information about the elasticity of the fluid." FLIM is already discussed in the Methods section on lines 268-269 and extensively in the "FLIM vs. ratiometry" subsection in the Supplementary Information.

Reviewer 2

I co-reviewed this manuscript with one of the reviewers who provided the listed reports. This is part of the Nature Communications initiative to facilitate training in peer review and to provide appropriate recognition for Early Career Researchers who co-review manuscripts.

Reviewer 3

The authors have taken some steps to tone down the claims in the paper based on its level of rigor. The method used is innovative and is likely to reveal much. Overall the manuscript is improved, but since the method is the key aspect the authors want to get across to the reader. They need to continue to be careful about the biological information provided by these studies given their low level of replication.

For example:
“A viscous mucus layer surrounding an ephyra of the upside-down jellyfish Cassiopea xamachana maintains a local microbiome while preventing direct contact with the animal.” In the abstract should be:
“A viscous mucus layer surrounding an ephyra of the upside-down jellyfish Cassiopea xamachana may maintain a local microbiome while preventing direct contact with the animal.”

> We agree and have changed the text in the abstract on line 40 to say “…may maintain a local microbiome…” as requested by the reviewer. We have made similar changes on lines 180 and 228.

I appreciate the authors efforts at improving the manuscript.
